# Chemical Profiling and Antioxidant and Anti-Amyloid Capacities of *Salvia fruticosa* Extracts from Greece

**DOI:** 10.3390/plants12183191

**Published:** 2023-09-06

**Authors:** Antonis Ververis, Sotiris Kyriakou, Kristia Ioannou, Paschalina S. Chatzopoulou, Mihalis I. Panayiotidis, Michael Plioukas, Kyproula Christodoulou

**Affiliations:** 1 Neurogenetics Department, The Cyprus Institute of Neurology and Genetics, Nicosia 2371, Cyprus; antonisv@cing.ac.cy (A.V.); kristia_311@hotmail.com (K.I.); 2 Department of Cancer Genetics, Therapeutics and Ultrastructural Pathology, The Cyprus Institute of Neurology and Genetics, Nicosia 2371, Cyprus; sotirisk@cing.ac.cy (S.K.); mihalisp@cing.ac.cy (M.I.P.); 3 Hellenic Agricultural Organization-DIMITRA, Institute of Plant Breeding and Genetic Resources, 57001 Thessaloniki, Greece; chatzopoulou@ipgrb.gr; 4 Department of Life and Health Sciences, School of Sciences and Engineering, University of Nicosia, Nicosia 2417, Cyprus; pmichael.gr@hotmail.com

**Keywords:** *Salvia fruticosa*, neuroprotection, plant extracts, Alzheimer’s disease, partitioning

## Abstract

An increasingly common ailment in elderly persons is Alzheimer’s disease (AD), a neurodegenerative illness. Present treatment is restricted to alleviating symptoms; hence, there is a requirement to develop an effective approach to AD treatment. *Salvia fruticosa* (SF) is a medicinal plant with a documented neuroprotective potential. To identify extracts of increased neuroprotectivity, we partitioned the methanolic extract of SF aerial parts from Greece into several fractions, by employing solvents of different polarities. The fractions were chemically identified and evaluated for their antioxidancy and anti-neurotoxic potential against amyloid beta peptides 25–35 (Aβ_25–35_). Carnosol and carnosic acid were among the prominent compounds, while all partitions showed significant antioxidant capacity, with the diethyl ether and ethyl acetate partitions being the most potent. These, along with the aqueous and the butanolic fractions, demonstrated statistically significant anti-neurotoxic potential. Thus, our findings further validate the neuroprotective potential of SF and support its ethnopharmacological usage as an antioxidant. The particular properties found define SF as a promising source for obtaining extracts or bioactive compounds, possibly beneficial for generating AD-related functional foods or medications. Finally, our results encourage plant extract partitioning for acquiring fractions of enhanced biological properties.

## 1. Introduction

One of the most widespread degenerative neurological conditions and the commonest dementia type among seniors is Alzheimer’s disease (AD). It is projected that there are almost fifty million patients with AD worldwide. With the ongoing increase and ageing of the global population, the number of sufferers is anticipated to double every five years, escalating further the vast burden AD imposes on humanity [1].

The main AD hallmarks are the build-up of the neurotoxic extracellular amyloid beta (Aβ) plaques and the development of intracellular neurofibrillary tangles in the brain, accompanied by oxidative stress, neuroinflammation, and neuron and synapse loss. Some of these features are interlinked and affect each other. In particular, oxidative stress is considered among the primary reasons for the Aβ deposits build-up, which further increases oxidative stress, causing additional cell damage, particularly to the neurons that are relatively more sensitive to oxidants [2].

Despite the dramatic consequences of AD, no therapy is currently available. Recent medication consists of drugs that only provide palliative and supporting treatment [3]. In 2021, the United States Food and Drug Administration provided accelerated approval for Aducanumab, an anti-amyloid drug targeting only the Aβ aggregated forms. However, it is still early to come to conclusions regarding Aducanumab’s effectiveness, and many scientists consider the approval premature because it was based on a surrogate endpoint [4]. Nevertheless, the need to develop novel effective drugs for AD remains, and targeting Aβ is the primary strategy being implemented in recent decades [5]. Due to their health-beneficial properties, medicinal plants constitute a large pool that can be investigated for the presence of a strong neuroprotective potential, which will oppose neurotoxicity caused by Aβ aggregation [6]. Proof of such a strong effect combined with an important antioxidant capacity can nominate certain medicinal plants as candidates for employment in the AD drug development industry and functional food market.

*Salvia fruticosa* Miller (SF), frequently cited as Greek sage, is an indigenous plant species of the eastern Mediterranean that belongs to the Lamiaceae family [7]. SF has shown various medicinal properties, such as anticancer [8], antibacterial [9], antiparasitic [10], antioxidant [11], hypoglycemic [12], anti-inflammatory [13], antiglycative [14], anticholinesterase [15], and neuroprotective [16,17]. Furthermore, it has been used as an herbal remedy throughout the Mediterranean countries to resolve a plethora of conditions, such as stomach ache, indigestion [18], diabetes [19], colds [20], depression, gingivitis, constipation, dysmenorrhea, hypotension [21], and diarrhea [22]. The presence of specific phytochemical constituents, with proven health-beneficial effects like 1,8-cineole, borneol, *p*-cymene, and camphor further supports and justifies the medicinal properties of SF [23]. 1,8-cineole has been the subject of various clinical trials of randomized, placebo-controlled, and prospective natures, to assess its efficacy, and presently, 1,8-cineole is an approved medication in Germany, for the treatment of bronchitis, rhinosinusitis, and several respiratory infections [24]. Borneol has also successfully undergone a randomized, double-blind, placebo-controlled clinical trial for the treatment of analgesia [25], while *p*-cymene has been subjected to a pharmacological study for the treatment of Fish Tapeworm Disease [26]. In addition, a combination of essential oils from *Salvia fruticosa*, *Coridοthymus capitatus*, and *Origanum dictamnus*, all of Cretan origin, has been generated to treat upper respiratory infections, but unfortunately, it did not show any significant statistical changes between the treated and the placebo group in a randomized, double-blind clinical trial, even though a slight improvement in the treated patients was observed [27]. However, a second observational study confirmed the potential of the referred essential oil combination to ameliorate the severity of the symptoms in the patients [28]. In another project, SF, among others, has been employed for the development of a substrate of polyphenols and flavonoids to prevent and treat peri-implant mucositis, with promising results in patients [29]. Finally, an open-label, single-arm, prospective clinical study implemented to assess the efficacy of a mixture of hydroxytyrosol and essential oils from several plant species, SF included, showed antidiabetic properties, such as reduced blood glucose [30].

We previously demonstrated a significant anti-neurotoxic activity for the SF dichloromethane and petroleum ether fractions, generated from aerial parts of the plant cultivated in Greece. These two fractions, along with the methanolic extract, presented an important antioxidant capacity as well. To our knowledge, this and another work showing neuroprotective properties in an SF infusion are the only available documentation of substantial anti-amyloid potential for SF [16,17].

Here, we investigated five novel SF fractions derived from the partitioning of the methanolic extract used in our previous work [16], with the expectation that partitioning would result in extracts of enriched biological activity. We assessed these extracts in terms of their antioxidant and anti-neurotoxic potentials, and additionally, we chemically characterized them to determine their chemical compounds. These efforts are important for the identification of SF mixtures or compounds with increased neuroprotective ability that can be advantageous for the development of a plant-based neuroprotection strategy.

## 2. Results

### 2.1. Salvia fruticosa Extracts Exhibit Noteworthy Total Phenolic (TPC) and Flavonoid (TFC) Contents

Each fraction presented considerable TFC (ranging from ~713 to 169 μg of catechin equivalents per gram of dry extract) and TPC (ranging from ~220 to ~86 of gallic acid equivalents per gram of dry extract), with the SFW1 (*Salvia fruticosa* initial aqueous extract) showing the highest levels in both categories, preceding SFEA (SF ethyl acetate extract) and SFDE (SF diethyl ether extract) in phenolics and SFW2 (SF remaining aqueous extract) in flavonoids. On the other hand, the SFB (SF butanolic extract) showed relatively low values in both categories. Furthermore, SFW1 exhibited the highest total soluble protein (TSPC) and total soluble sugar (TSSC) contents, whereas SFEA and SFDE showed near-zero values for these categories (Table 1).

### 2.2. Salvia fruticosa Extracts Are Rich in Carnosic Acid and Carnosol

Moreover, we identified and quantified some of the commonest polyphenolics by employing UPLC-MS/MS with the use of external standards. For the detection of the most prevalent polyphenolic and flavonoid compounds selected, an ion recording (SIR) approach was employed (Figure S1). The results showed that SF is especially abundant in carnosic acid and carnosol, particularly the ethyl acetate and butanolic partitions. Rosmarinic acid, chlorogenic acid, gallic acid, ferulic acid, caffeic acid, coumarin, quercetin, apigenin, and luteolin were found specifically enriched in the SSW1 extract (Table 2). In general, the derivatives of the benzoic acid were found slightly enriched in SFW1 and SFEA, the derivatives of gallic acid, cinnamic acid, coumarin, flavone, and flavonol were at higher concentrations in SFW1, and finally, diterpenes were substantially greater in SFEA and SFB in comparison to the other partitions.

### 2.3. Salvia fruticosa Fractions Demonstrate a Considerable Antioxidant Potential

#### 2.3.1. DPPH· Assay

We assessed the antioxidant potency of the five SF fractions with the employment of three different assays. Radical scavenging capacity, defined as *EC*_50_ (the quantity of fraction needed to lower the starting DPPH· (stable radical 1,1-diphenyl,2-picrylhydrazyl) concentration by 50%), fluctuated between 0.08 to 0.47 mg dry extract/mg DPPH· (Table 3). All five fractions exhibited respectable antiradical activity, especially the SFDE fraction with an antiradical efficiency (AE) two times the value of Trolox (AE_Trolox_(DPPH·): 5.59), which is used as a standard antioxidant because of its high radical scavenging activity [31].

#### 2.3.2. FRAP Assay

Similarly, in the FRAP (ferric reducing activity power) assay, the SF extracts demonstrated a strong antioxidant potential, as shown in Table 3. The extracts showed a strong ability to neutralize free radicals, ranging from 1165 to 3880 μmol ascorbic acid per g (AAE/g) partition and 1237–4229 μmol Trolox per g (TEAC/g) partition. According to FRAP, the most robust antioxidant was the SFEA.

#### 2.3.3. DCFDA Assay

**Figure 1:**
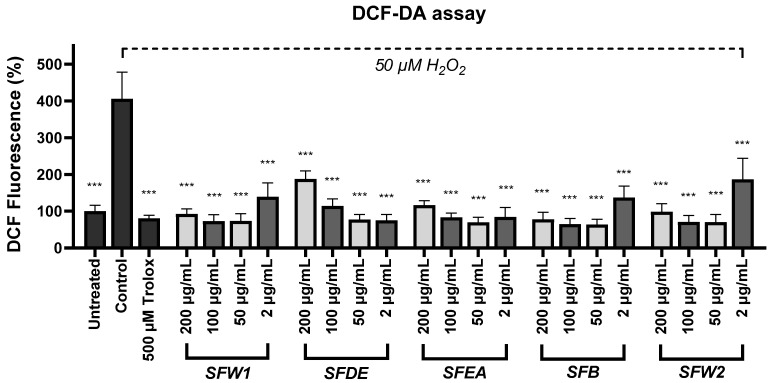
The five partitions of *Salvia fruticosa* methanolic extract exhibit an important antioxidant potential in SH-SY5Y cells in response to oxidative stress triggered by H_2_O_2_. The DCFDA test was employed to assess the reactive oxygen species activity in cells upon each condition. The standard error of the mean for five independent experiments is shown by error bars. *** designates statistical importance at *p* < 0.001, against control cells that received a 50 μM H_2_O_2_ treatment.

Finally, the DCFDA (2′,7′–dichlorofluorescin diacetate) cellular reactive oxygen species (ROS) detection assay was conducted to evaluate the antioxidancy of the fractions in SH-SY5Y cells. One-way analysis of variance was utilized to identify variations among group means that were statistically significant (F (22, 92) = 7.445, *p* < 0.001). Post hoc Dunnett’s multiple comparison tests to compare the control with each different extract’s treatment (Table S1) confirmed the antioxidant potential of all five SF fractions, which significantly decreased the oxidative stress levels in cells treated with H_2_O_2_ (Figure 1). All the fractions were strikingly effective, since they reduced oxidative stress in similar and even lower levels in comparison to those observed after treatment with 500 μΜ Trolox. The optimal concentration exhibiting this type of activity in the SH-SY5Y cells was 50 μg/mL for SFW1, SFEA, SFB, and SFW2, and 2 μg/mL for SFDE. The reduction in antioxidant efficiency in the 200 μg/mL of SFDE prompted us to investigate whether this effect would be completely lost in even higher concentrations of the extracts. We found that the SFDE at 800 and 400 μg/mL was not effective, in contrast to all the other fractions that retained their antioxidancy in that range as well (Figure S2).

### 2.4. Salvia fruticosa Fractions Are Cytotoxic above a Concentration Limit

**Figure 2:**
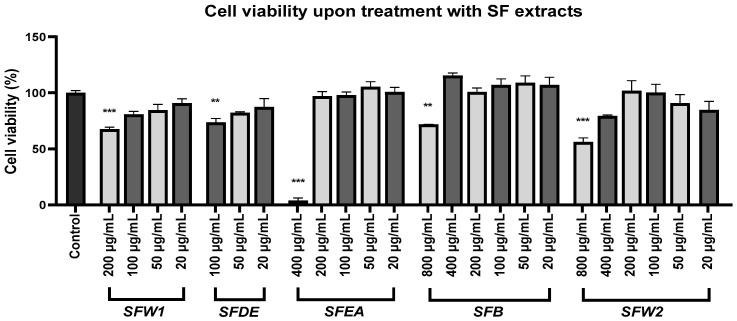
The five *Salvia fruticosa* methanolic partitions show cytotoxicity above a concentration limit in SH-SY5Y cells. The MTT assay was employed for the evaluation of cell viability. The standard error of the mean of four separate assays is represented with the error bars. In comparison to untreated cells (control), ** and *** designate statistical importance at *p* < 0.01 and *p* < 0.001 correspondingly.

Before evaluating the neuroprotective potential of the various SF fractions, we first looked into the extracts’ cytotoxicity in different concentrations for 48 h, to avoid treating SH-SY5Y cells with concentrations that are harmful to them. As anticipated, treatments with relatively high concentrations of the fractions (SFW1 ≥ 200 μg/mL, SFDE ≥ 100 μg/mL, SFEA ≥ 400 μg/mL, SFB ≥ 800 μg/mL, SFW2 ≥ 800 μg/mL) significantly decreased SH-SY5Y viability (Figure 2). These differences were determined statistically with the employment of a one-way analysis of variance (F (24, 75) = 21.90, *p* < 0.001), then Dunnett’s post hoc test was employed for the purpose of comparing the cell viability in the untreated control cells with the cell viability after each extracts’ treatment (Table S2).

### 2.5. Various Salvia fruticosa Fractions Exhibit Neuroprotective Activity

**Figure 3:**
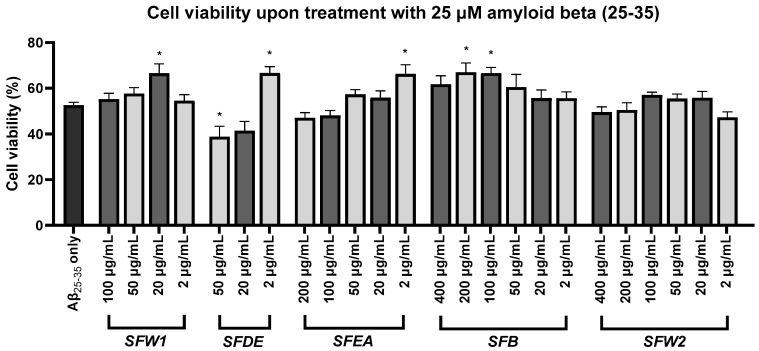
Neuroprotective potential of the five *Salvia fruticosa* methanolic partitions on amyloid-beta-caused cell viability reduction in SH-SY5Y cells. The MTT assay was employed for the evaluation of cell viability. The standard error of the mean of five separate studies is represented by the error bars, and * designates statistical significance at *p* < 0.05, upon comparison with cells treated with 25 μM Aβ_25–35_ only.

To assess the neuroprotective potential of the fractions, we treated the cells with 25 μM Aβ_25–35_ aggregates for 48 h, which resulted in a ~48% cell viability reduction. Four of the SF fractions under investigation exhibited a neuroprotective capacity, since pre-treating the cells with specific concentrations of them significantly reduced the viability decrease that the Aβ_25-35_ aggregates caused. Specifically, the SFW1 at 20 μg/mL, the SFDE and the SFEA at 2 μg/mL, and the SFB at 200 and 100 μg/mL showed a statistically significant neuroprotective potential against Aβ_25–35_ toxicity (Figure 3). These fractions at the above concentrations restored cell viability at ~66% relative to the untreated control cells. Only the SFW2 fraction did not show any significant neuroprotective capacity at the investigated concentrations. One-way analysis of variance (F (24, 100) = 6.011, *p* < 0.001) was conducted, and then Dunnett’s post hoc analysis was used to compare each condition to the control, to identify the statistically important differences in cell viability (Table S3).

## 3. Discussion

### 3.1. Phytochemical Composition

*Salvia fruticosa* is a medicinal plant, known for its neuroprotective properties [16,17]. Here, we elaborated further on this potential. Specifically, we obtained five SF fractions with the use of different solvents (diethyl ether, ethyl acetate, butanol, and water), that are partitions of the methanolic extract used in our previous work [16]. These five partitions differ in their secondary metabolite content, as shown by the unique polyphenolic fingerprint of each fraction. It is expected that plant extracts obtained by different solvents possess specific phytochemical profiles, associated possibly with their bioactivity, and hence medicinal properties [32]. We showed that all five SF fractions are strong antioxidants, while four of them exhibit neuroprotectivity as well.

The investigation of the TPC and TFC of the fractions showed them at relatively high levels in the different fractions, in comparison to previous SF studies [8,13,16,33]. The plants’ genotype, the developmental stage, the environmental conditions at the growing site, the season of harvesting, and the presence of environmental stressors, as well as the extraction process and the solvent used, are parameters that affect the phytochemical profile and the bioactivity of the extract [33,34,35,36]. Therefore, the specific genotype and the growing conditions of the plants used in this study favour the extensive presence of phenolics and flavonoids.

### 3.2. Antioxidant Capacity

DPPH^.^, FRAP, and DCFDA assays revealed a strong antioxidant potential of all fractions, which seem to surpass Trolox in many cases. Notably, DPPH⋅ and FRAP assays presented the methanolic partitions SFDE and SFEA with greater antioxidant capacity in comparison to the methanolic extract that they originate from [16], showing an improved allocation of the plant material due to partitioning. The significant presence of flavonoids and phenolics, which are regarded as two primary categories of plant antioxidants, probably contributes to the antioxidant effect [37], even though previous studies have shown that a strong antioxidant potential in SF does not necessarily correlate with high TPC or TFC [13,16]. Finally, the identification of substances with antioxidant properties in the fractions, such as carnosol, carnosic acid, rosmarinic acid, quercetin, chlorogenic acid, ferulic acid, apigenin, and luteolin, confirm our findings regarding the antioxidant potential of the SF fractions [38,39,40,41,42,43].

The DCFDA assay showed that there is an optimal concentration range in which each extract expresses better its antioxidancy. For the majority of the fractions, this range was between 50 and 100 μg/mL, except for the SFDE, where it was observed between 2 and 50 μg/mL. In every partition, the antioxidant effect seems to fade away at smaller concentrations (≤2 μg/mL), with the only exception being, again, the SFDE, which showed its higher antioxidancy level at 2 μg/mL. We believe that we would have observed an antioxidant capacity reduction in the smaller concentrations of the SFDE if we had used a broader range that would extend below 2 μg/mL. After all, as the concentration of the extract drops, the compounds that confer antioxidancy are diluted; thus, it is common sense to expect that the extract’s antioxidant activity will be weakened beyond a certain point. The extracts in concentrations above the optimal range retain their antioxidancy, even with a seemingly small decrease in some of them, except for the SFDE, in which an important reduction was observed that led to the loss of antioxidancy in concentrations above 200 μg/mL (Figure S2). Similar phenomena have been observed in works by others in different plants, as well [44,45]. We can assume that this may be happening because of possible aggregation phenomena of the antioxidant compounds taking place in higher concentrations of the methanolic partitions [46]. Additionally, the phytochemical characterization showed *trans*-cinnamaldehyde to be relatively enriched in SFDE (Table 2). *Trans*-cinnamaldehyde has been shown to enhance oxidative stress in *Arabidopsis thaliana* by increasing the benzoic acid levels [47], to induce developmental neurotoxicity in zebrafish by enhancing oxidative stress [48], and to reduce oxidative metabolism in murine myotubes [49]. Thus, it may play a role in the loss of antioxidancy observed after treatment with SFDE in relatively high concentrations, in which the other partitions retain their antioxidant potential. Conversely, other reports have shown cinnamaldehyde to protect from the oxidative stress consequences in various cell lines and rat and mouse models [50,51]; hence, future research is required to fully elucidate the mode of action of this substance. Overall, cinnamaldehyde in high dosages may exert toxicity, while in non-toxic concentrations it expresses a health-beneficial potential [52].

### 3.3. Anti-Neurotoxic Potential

The neuroprotectivity investigation showed that the SFW1, SFDE, SFEA, and SFB possess a statistically significant neuroprotective capacity against amyloid beta toxicity. Only the SFW2 extract did not ameliorate the Aβ_25–35_ neurotoxicity. These four fractions that exhibit neuroprotective properties are partitions of the methanolic extract, used in Ververis et al., 2020 [16]. In that work, the methanolic extract did not exhibit a statistically important anti-neurotoxic activity, in contrast with four of its partitions that showed this type of potential in the present study. This finding is very important, since it shows that partitioning specific extracts can lead to more effective utilization of the plant material, to fully uncover the various potentials of the plant. Together with our previous work regarding the presence of similar properties in the extracts derived with the use of petroleum ether and dichloromethane [16], these results further confirm SF as a plant species with a high neuroprotective capacity.

The four SF extracts that showed neuroprotectivity expressed this ability at specific concentrations: the SFW1 at 20 μg/mL, the SFDE and the SFEA at 2 μg/mL, and the SFB demonstrated this property at 200 and 100 μg/mL. These variations are possible due to the qualitative and quantitative differences in the chemical constitution of these fractions. Nevertheless, neuroprotectivity tends to fade away in smaller concentrations (Figure 3), whereas in relatively higher concentrations the extracts cause cytotoxicity (Figure 2). Furthermore, in cells treated with Aβ_25–35_ and 50 μg/mL SFDE, cell viability dropped further in comparison to cells treated with Aβ_25–35_ only (Figure 3). At this concentration, SFDE dropped cell viability in a non-statistically significant way (Figure 2), and it is possible that the simultaneous presence of Aβ_25–35_ and 50 μg/mL SFDE in cells amplifies toxicity and further reduces cell viability, in relation to cells treated with Aβ_25–35_ only. Previous works have demonstrated that SF extracts and essential oils induce cytotoxicity above a concentration limit that is dependent on the cell line under treatment [16,53,54]. Relatively high concentrations of SF extracts have been demonstrated to reduce mitotic index in human lymphocytes and inhibit cell division in A375 cells [55,56]. In addition, the methanolic extract used in this work has been shown to trigger apoptosis in A375 cells, mainly by activating the extrinsic signalling pathway [8]. Finally, some of the main SF constituents identified here, such as carnosol and rosmarinic acid, demonstrate cytotoxic activity as their concentration increases, whereas carnosic acid causes cell cycle arrest in various cell types [56,57,58].

SF’s mechanism to exert anti-neurotoxicity on amyloid-beta-treated cells is certainly dependent on its bioactive compounds index and the synergistic, additive, and antagonistic interactions between the various compounds [59]. The presence of substances with neuroprotective properties in the SF methanolic partitions, like carnosic acid, carnosol, rosmarinic acid, apigenin, quercetin, luteolin, and chlorogenic acid is likely playing an important role in this effect’s unfolding [60,61,62,63,64,65]. In particular, carnosic acid, which is the most enriched substance to have been identified in the SF fractions at our disposal, inhibits the phosphorylation of NMDAR2b (*N*-methyl-D-aspartate receptors subtype 2b) that results in reduced cell death in an AD model system utilizing SH-SY5Y cells. Additionally, carnosic acid reverses synaptic deficits by boosting the expression of synaptophysin, PSD-95 (postsynaptic density protein-95), and BDNF (brain-derived neurotrophic factor) [60]. Moreover, carnosic acid decreases Aβ release by blocking the CEBPβ-NFκB signalling pathway, which is associated with brain damage and degeneration [66]. Carnosol, the other abundant substance found in the SF partitions, improves protein homeostasis by stimulating molecular chaperones and controlling the MAPK (mitogen-activated protein kinase) pathway, and among other things, enhances mitochondrial homeostasis by triggering relevant genes, and reduces neuron damage by inducing Notch signalling [61]. Furthermore, a recent study has indicated that SF infusion, whose main constituent is rosmarinic acid, inhibits CK1-δ (casein kinase 1δ), BACE-1 (β-secretase), and GSK-3β (glycogen synthase kinase 3β), which are proteins whose aberrant expression has been associated with Aβ toxicity and neurodegeneration. The inhibition of these proteins by SF may explain, at least partly, its neuroprotective activity [17,67,68,69].

### 3.4. Medicinal Perspective of Salvia fruticosa Fractions

Oxidative stress and the development of Aβ plaques are two processes that likely affect positively each other. Specifically, in AD, oxidative stress promotes Aβ plaque development, while Aβ plaques intensify oxidative stress [2,70,71]. Thus, through its antioxidant potential, further confirmed here, SF can protect the cells from the toxicity caused by Aβ_25–35._ Moreover, it was shown in a previous study that an essential oil derived from SF ameliorated neuronal death caused by hydrogen peroxide [72]. Hence, a good strategy for fighting AD may be the development of drugs with considerable antioxidant and neuroprotective properties, and since SF fractions, and specifically, SFW1, SFDE, SFEA, and SFB, possess both of these potentials, they can be considered proper candidates to be further investigated as plant-based medicine in the search for an effective treatment for AD. The most promising of these four extracts are SFDE and SFEA, which present similar anti-amyloid activity as the rest, but also an enormous antioxidant ability, which is relatively high in comparison to other medicinal plants [73]. Furthermore, their anti-amyloid capacity is similar to extracts from other medicinal plants that are considered neuroprotective, as well [74,75,76]. However, SFDE and SFEA do not completely restore the viability of the cells, as other individual compounds or specific extracts from other plants do [77,78].

Nonetheless, future studies are needed in other systems, such as in vivo, to confirm and fully uncover the neuroprotectivity of these SF fractions in living organisms. AD mouse models can serve as the next step for the evaluation of any cognitive improvement that may occur after treatment with the above SF fractions, and the identification of any possible side effects. Cell lines are generally considered a proper tool for the initial testing in drug testing; however, they restrict the evaluation of important pharmacokinetic parameters, and, hence, further in vivo experimentation is required [79]. This will provide valuable information for the determination of the degree to which SF can be involved in the fight against AD, either in drug development or as a functional food and supplement.

Conclusively, we have shown a significant antioxidant and anti-amyloid potential for the majority of the SF fractions, especially for SFDE and SFEA. These fractions consist of various phytochemicals and can potentially serve a multitarget approach against multifactorial AD. Additionally, we have exhibited the importance of partitioning plant extracts to acquire mixtures of increased biological activity.

## 4. Materials and Methods

### 4.1. Chemicals

Every solvent and reagent utilized here was obtained from Sigma-Aldrich (Taufkirchen, Germany), except for DMSO, which was supplied by Santa Cruz (Heidelberg, Germany). The analytical standards employed were purchased from Extrasynthese (Lyon, France), except apigenin, luteolin, neochlorogenic acid, 4-*O*-caffeoylquinic acid, carnosol, and carnosic acid, which were supplied by Adooq Bioscience (Irvine, CA, USA).

### 4.2. Plant Material

The Institute of Plant Breeding and Genetic Resources (IPGRB) of the Hellenic Agricultural Organization “DIMITRA” provided the plant material. More specifically, aerial parts of SF cultivated plants in the experimental field of IPGRB, Thessaloniki, Greece, were collected in full flowering, as previously described [16], and dried at ambient temperature.

### 4.3. Plant Extracts

A fixed weight (56.17 g) of SF aerial parts was contained in a Soxhlet device 0.6 L and was, consecutively, extracted with petroleum ether and dichloromethane for 22 h and 26 h, respectively. The plant material, after defatting, was exhaustively extracted for 51 h with methanol in a Soxhlet apparatus, and the obtained fraction was vacuum-evaporated until dry. The temperature of the Soxhlet apparatus was set at the boiling point of every solvent. The plant material was then separated using 700 mL of 75 °C water, while the produced extract (SFW1) was dried up. The dried remaining methanolic extract was dissolved in 300 mL of hot water heated to 75 °C, filtered, and partitioned using solvents of increasing polarity (diethyl ether, ethyl acetate, and *n*-butanol—7-fold of 20 mL, 22-fold of 15 mL, and 10-fold of 15 mL, correspondingly). All organic layers from each of the above three solvents (SFDE, SFEA, and SFB, correspondingly) were concentrated to dryness under reduced pressure and the remaining aqueous extract (SFW2) was then finally gathered. The aforementioned extracts were made at room temperature. The fractions were dissolved in DMSO before use for the various experiments conducted in cells.

### 4.4. DPPH· Assay

Radical scavenging activity against the DPPH^.^ was executed as previously reported [80]. In a nutshell, 20 μM DPPH· mother solution was generated in methanol. Then, 975 μL of DPPH^.^ was combined with 25 μL of SF fraction, vortexed, and incubated at RT. The reduction in absorbance was captured at 517 nm. Trolox served as a positive control. The potential for antioxidant action for every fraction was quantified as the amount of fraction demanded to lower the starting DPPH^.^ levels by half (*EC*_50_). The antiradical efficiency (*AE*) is inversely proportional to *EC*_50,_ since it is determined according to Relation (1).

(1) $$AE=\frac{1}{{EC}_{50}}$$

### 4.5. FRAP Assay

The FRAP test was conducted in accordance with other reports [74,81]. Initially, fresh FRAP solution was made by combining (10:1:1: *v*/*v*/*v*) 300 mM acetate buffer (pH 3.6), 10 mM TPTZ in 40 mM HCl, and 20 mM iron(III) chloride solution. 0.2 mL of SF fraction was added to 1.8 mL of FRAP solution and the reaction was left to sit for 10 min at RT. The absorbance was captured at 593 nm. Using Trolox and ascorbic acid standard curves, the ferric reducing power was represented as Trolox equivalent antioxidant capacity (TEAC mol/g sample), and as ascorbic acid equivalents (AAE mol/g sample), respectively.

### 4.6. Estimation of Total Phenolic Content

The Scalbert et al. approach, with a few minor modifications, was used to calculate the TPC of the plant extract, as previously mentioned [16,82]. Briefly, in a test tube, 2.5 mL of a 10-fold diluted Folin–Ciocalteu’s phenol reagent, 2 mL of a 7.5% sodium carbonate solution, and 0.5 mL of the extract were combined and rapidly shaken. Using a spectrophotometer, absorbance at 760 nm was measured after 30 min at RT. Using a gallic acid standard curve, the TPC of each partition was calculated and presented as gallic acid equivalents (GAE mg/g sample). The results of the analyses were expressed as averages with SDs after being executed in triplicate.

### 4.7. Estimation of Total Flavonoid Content

The TFC of each partition was assessed with the aluminium chloride colorimetric approach with a few minor alterations, as previously documented [16,83]. Briefly, after dilution with 120 μL of 0.5 M methanol, 40 μL of each fraction was added to 20 μL of aluminium trichloride (10% *v*/*v*) and 20 μL of sodium acetate. The resulting mixes were placed in a darkened room at room temperature for 40 min before their absorbance was captured at 415 nm. TFC was presented as μmol of catechin hydrate equivalents (CE) per gram of dried fraction. Analyses were executed in triplicate and are displayed as average results with SDs.

### 4.8. Calculation of Total Soluble Sugar Content

With several minor modifications, the TSSC determination was conducted as previously described [84]. In a nutshell, 200 μL of each SF reconstituted extract was dehydrated by concentrated sulfuric acid (150 μL), followed by mixing with 30 μL of 5% phenol. The mixtures were warmed for five minutes at 90 °C. Then, the solutions were given time to settle at RT. Utilizing an LT4500 microplate reader to detect absorbance at 490 nm, the findings were expressed as nmol of mannose equivalents/g of dried extract.

### 4.9. Total Soluble Protein Content Calculation

The bicinchoninic acid protein assay kit (Thermo Scientific, Waltham, MA, USA) was employed to estimate the amount of TSPC in the sample. On an LT4500 reader, the absorbance was read at 562 nm. Based on a standard curve for bovine serum albumin (BSA), the TSPC was assessed. The content of proteins was defined as mg of protein per gram of dried extract.

### 4.10. Establishing Standards and Samples

The initial solutions for the reference standards were prepared at a 1000 ppm concentration in either a 1:1 methanol/acetonitrile or 1:1 methanol/water combination. Ice-cold methanol was used to carry out additional dilutions. Every sample was stored in the dark and protected from light since polyphenols (mostly flavonoids) are light-sensitive. All solutions were filtered through a membrane using mixed cellulose esters (MCE) with a 0.22 μm pore size prior to UPLC-ESI-MS/MS analysis.

### 4.11. Ultra-Performance Liquid Chromatography–Tandem Mass Spectrometry (UPLC-MS/MS)

#### 4.11.1. Liquid Chromatography (LC) Conditions

The chromatographic separation was performed on a Waters Acquity UPLC system (Waters Corp., Milford, MA, USA), with an autosampler chamber, two pumps, and a degasser, employing an ACQUITY UPLC BEH C18 (100 × 2.1 mm, particle size: 1.7 μm) column, warmed to 30 °C, and eluted as before presented with a few adjustments [85]. Acetonitrile (eluent A) and a solution of formic acid 0.1% (*v*/*v*) (eluent B) were used to elute the mobile phase. Linear gradient settings ranging from 5–100% A (0–4 min), 100–90% A (4.0–4.1 min), 90% A (4.1–5 min), 90–5% A (5–5.01 min), and 5% A (5.1–8 min) were used with a flow rate of 0.3 mL/min. The amount of injection was 10 μL, and the autosampler had been configured at 4 °C.

#### 4.11.2. MS/MS Conditions

A Xevo Triple Quatrable (QqQ) mass spectrometer detector (Waters Corp.) was employed in the MS/MS studies and operated in positive or negative electron spray ionisation mode (ESI^±^). The analytes quantification was performed by employing multiple reaction monitoring (MRM) transitions. Before the sample analysis, each standard was manually tuned to achieve the optimum MRM conditions at 1 ppm concentration (Table S4, Figure S3). To get the greatest signal level, 3.0 kV, the ideal tuning settings were as follows: 36 V at the cone, 150 °C at the source, 500 °C at the disolvation, 1000 L/h at the source disolvating gas flow, and 20 L/h at the gas flow. Ultra-high-purity argon was employed as a collision gas, and high-purity nitrogen gas served as the drying and nebulizing gas. Data were gathered and processed using the MassLynx program (version 4.1, Waters Co., Milford, MA, USA).

#### 4.11.3. Optimization of UPLC and MS Conditions

To address the generation of peaks with optimum sharpness and symmetry, multiple parameters were adjusted, including those of elution mode, mobile phase, and flow rate. More specifically, the elution was accomplished with various solvent combinations, including those of acetonitrile/water and methanol/water at different ratios, with none of these yielding symmetrical peaks. However, acidification of water with formic acid (0.1% *v*/*v*) afforded peaks of improved symmetry and sharpness. In addition to this, the fragmentation of the analytes was also improved, since formic acid facilitates molecular ionisation. Furthermore, the optimum separation was performed on an ethylene-bridged hybrid (BHE) column pre-warmed to 40 °C.

#### 4.11.4. Method Validation

The guidelines set out by the International Conference on Harmonization were adhered to [86]. Parameters such as linearity, limits of detection (LOD) and quantification (LOQ), precision, and accuracy were found and evaluated for each analyte (Table S5). A linear regression equation of response peak areas as an indicator of the different standards’ concentrations, which ranged from 0 to 500 ppb, was used to draw the resulting standard curves for the standards (Table S5). Each of the compounds under investigation showed strong linearity, with a coefficient of correlation (R^2^) greater than 0.99 (Table S5). Finally, the percentage of recovery (Table S5) was used to evaluate the repeatability of the analytical process. In this regard, each standard solution was added to a different proportion of *S. fruticosa*. It was possible to obtain the measurements of the minimum of six repetitions from spike samples that were created in triplicate. The percentage of recovery was computed using Relation (2), where *A* is the final quantity identified, *A*_0_ is the original quantity, and *A_a_* is the additional quantity:

(2) $$\%\;recovery=\left[\frac{\left(A-A_{0}\right)}{A_{a}}\right]\times 100$$

The average rate of recovery of all substances discovered, which ranged from 86.3% to 102.6%, served as evidence of the accuracy and repeatability of the aforementioned methodology.

#### 4.11.5. Linearity, Accuracy, and Precision of the Methodology

To evaluate the specificity and selectivity of the analytical procedure, the LOD and LOQ values were derived utilizing the signal-to-noise (S/N) ratios set at 3 and 10, correspondingly. The LOD and LOQ ranges for polyphenolic compounds were 0.11–97.28 ppb and 0.30–294.81 ppb, correspondingly. Ferulic acid ethyl ester’s sensitivity to detection was substantially greater when compared to the other listed substances that had been ionised in the same way, according to the values in Table S5, where ferulic acid ethyl ester had the smallest LOD and di-hydrocaffeic acid had the greatest. It was therefore feasible to determine how similar the several samples were to each other within the same uniform by determining the percentage of relative standard deviation (% RSD). To estimate the inter-day accuracy and intra-day accuracy of six duplicated samples of similar concentrations over the course of one day and six consecutive days, respectively, the % RSD was determined. For the polyphenolic compounds in particular, the intra- and inter-day RSD readings varied from 0.70 to 4.68% (Table S5).

### 4.12. Peptides Preparation

To allow for the formation of aggregates, Aβ_25–35_ peptides (Genscript, Piscataway, NJ, USA) were dissolved at a concentration of 1 mM in sterile distilled water, as previously reported [16]. Prior to application, the peptides were stored in portions at −20 °C. 

### 4.13. Cell Culture

The ATCC (Manassas, VA, USA) supplied the human neuroblastoma SH-SY5Y cells, which were then grown in DMEM medium along with 10% fetal bovine serum, 5% horse serum, 2 mM glutamine, 50 U/mL penicillin, and 50 mg/mL streptomycin (Biosera, Nuaille, France), as previously reported [16]. Cell culture took place in an incubator set at 5% CO_2_ and 37 °C.

### 4.14. DCFDA Assay

2′,7′–dichlorofluorescin diacetate (DCFDA) is a fluorogenic dye used to evaluate the concentration of reactive oxygen species [87]. A total of 25,000 SH-SY5Y cells were introduced in every well of a black 96-well plate (SPL Life Sciences, Naechon-myeon, Republic of Korea). The following day, cells were treated with specific concentrations of SF extracts while being exposed to 50 μM hydrogen peroxide, after being treated with 20 μM DCFDA for 45 min at 37 °C in the absence of light. Fluorescence was determined in a microplate reader at Ex/Em = 485/535 nm. The typical antioxidant used was Trolox (500 μΜ). Five independent tests were carried out, and the assay was done in triplicate.

### 4.15. MTT (3-[4,5-dimethylthiazol-2-yl]-2,5 diphenyl tetrazolium bromide) Assay

MTT assay was conducted to assess cell viability [88]. In 96-well plates, 20,000 SH-SY5Y cells were introduced into every well. One day later, cells were incubated with the SF fractions for 2 h, and then Aβ_25–35_ was added to the mixture, wherever needed, at an endpoint concentration of 25 μM for two days. Next, the cells were incubated for 4 h at 37 °C in a growth medium without phenol red that was supplemented with 45 μg/mL MTT. The culture material was then aspirated, and each well received 150 μL of DMSO. On an orbital shaker, the plate was shaken for 15 min while being covered with foil, and then, at 590 nm, the absorbance was measured. Using Equation (3), the proportion of cell viability was computed.

(3) $$\%\;cell\;viability=\left[\frac{mean(OD\;treatetd\;cells-OD\;blank)}{mean(OD\;control-OD\;blank)}\right]\times 100$$

The assay was performed in triplicate. Regarding the cytotoxic effect of SF fractions, four independent tests were carried out, and five independent studies looked at their neuroprotective effect.

### 4.16. Statistical Analysis

Data are displayed as mean values ± standard deviation or mean values ± standard error of the mean. Statistical analyses were conducted by one-way ANOVA with Tukey’s (Section 4.6, Section 4.7, Section 4.8, Section 4.9 and Section 4.11) or Dunnett’s test (Section 4.14 and Section 4.15) for multiple comparisons with the use of GraphPad Prism 9.5.1.

## 5. Conclusions

The present study demonstrated additional evidence confirming the neuroprotective potential of SF on Aβ neurotoxicity, in addition to its strong antioxidant capacity. These two traits can be particularly useful in designing and developing a plant-based AD-fighting strategy. The study also showed that partitioning can help in unveiling or maximizing the desired effects that plant extracts may possess. Further investigation of the extracts in vivo is required to acquire a complete understanding of their neuroprotective capacity.

## Figures and Tables

**Table 1:** Assessment of total phenolic, flavonoid, soluble protein, and soluble sugar contents of the partitions of *S. fruticosa* methanolic extract.

	SFW1	SFDE	SFEA	SFB	SFW2
Total Phenolic Content (μg of gallic acid eq/g of dry extract) Linear Range: 0–500 μg/mL; y = 0.0052x + 0.018, R^2^ = 0.9981	219.95 ± 3.32 ^e^	159.98 ± 4.95 ^c^	189.45 ± 7.65 ^d^	86.21 ± 2.21 ^b^	50.98 ± 1.88 ^a^
Total Flavonoid Content (μg of catechin eq/g of dry extract) Linear Range: 0–500 μg/mL; y = 0.0028x − 0.36, R^2^ = 0.9991	713.26 ± 8.15 ^d^	226.14 ± 13.48 ^b^	168.21 ± 14.14 ^a^	179.21 ± 6.35 ^a^	654.21 ± 5.11 ^c^
Total Soluble Protein Content (mg of BSA eq/g of dry extract) Linear Range: 0–2 mg/mL; y = 0.6851x + 0.1345, R^2^ = 0.9958	129.67 ± 5.47 ^d^	n.d.	0.32 ± 0.02 ^a^	5.98 ± 0.98 ^b^	66.21 ± 2.25 ^c^
Total Soluble Sugar Content (nM of mannose eq/g of dry extract) Linear Range: 0–100 nM; y = 0.01645x + 0.1578, R^2^ = 0.9999	197.13 ± 6.32 ^c^	n.d.	n.d.	62.31 ± 4.87 ^a^	85.61 ± 4.23 ^b^

The data describe means ± standard deviation (SD) of a minimum of three independent experiments. Means and SDs preceded by distinct letters in a category significantly vary, as indicated by Tukey’s test (*p* < 0.05). The initials n.d. represent data for which the associated signals were not detectable.

**Table 2:** Quantitative data displaying the phytochemical composition of the five *S. fruticosa* fractions (SFW1, SFB, SFEA, SFDE, and SFW2).

	SFW1	SFDE	SFEA	SFB	SFW2
Benzoic acid derivatives (μg/g of dry extract)					
*m*-hydroxy benzoic acid	3.59 ± 0.09 ^c^	0.89 ± 0.06 ^a^	1.54 ± 0.09 ^b^	4.19 ± 0.16 ^c^	6.18 ± 0.04 ^d^
*p*-hydroxy benzoic acid	n.d.	1.21 ± 0.11 ^a^	14.98 ± 0.81 ^c^	5.26 ± 0.11 ^b^	n.d.
Protocatechuic acid	24.59 ± 1.12 ^c^	0.87 ± 0.06 ^a^	3.14 ± 0.01 ^b^	n.d.	n.d.
Vanillin	14.59 ± 1.03 ^c^	n.d.	23.36 ± 1.56 ^d^	5.69 ± 0.01 ^b^	0.21 ± 0.01 ^a^
*p*-hydroxy benzaldehyde	16.69 ± 1.01 ^b^	n.d.	1.13 ± 0.01 ^a^	n.d.	n.d.
Gentisic acid	30.36 ± 2.51 ^c^	n.d.	3.48 ± 0.12 ^b^	1.11 ± 0.01 ^a^	4.16 ± 0.02 ^b^
Gallic acid derivatives (μg/g of dry extract)					
Gallic acid	98.15 ± 4.89 ^d^	12.21 ± 1.01 ^a^	17.11 ± 0.98 ^b^	n.d.	25.59 ± 1.12 ^c^
Ethyl gallate	3.69 ± 0.26 ^c^	0.36 ± 0.02 ^a^	8.36 ± 0.36 ^d^	0.49 ± 0.01 ^b^	n.d.
Syringic acid	7.89 ± 0.69 ^c^	0.24 ± 0.04 ^a^	13.21 ± 1.07 ^d^	0.36 ± 0.01 ^b^	n.d.
Ellagic acid	10.21 ± 0.09 ^b^	n.d.	n.d.	0.13 ± 0.01 ^a^	n.d.
Cinnamic acid derivatives (μg/g of dry extract)					
Ferulic acid	159.26 ± 10.21 ^d^	19.29 ± 1.36 ^b^	2.21 ± 0.63 ^a^	63.12 ± 2.31 ^c^	n.d.
Ferulic acid ethyl ester	69.98 ± 2.45 ^b^	13.39 ± 1.02 ^a^	79.89 ± 4.94 ^c^	n.d.	n.d.
Ferulic acid methyl ester	n.d.	0.020 ± 0.001 ^a^	16.26 ± 1.35 ^b^	n.d.	n.d.
Caffeic acid	78.98 ± 3.65 ^d^	14.23 ± 1.11 ^b^	23.32 ± 1.59 ^c^	5.59 ± 0.04 ^a^	n.d.
Dihydro caffeic acid	1.36 ± 0.65 ^a^	3.91 ± 0.14 ^b^	2.21 ± 0.10 ^a^	n.d.	n.d.
*trans*-cinnamaldehyde	n.d.	6.24 ± 0.37 ^c^	1.21 ± 0.01 ^b^	0.020 ± 0.001 ^a^	n.d.
*trans*-cinnamyl alcohol	6.69 ± 0.41 ^b^	0.67 ± 0.01 ^a^	n.d.	n.d.	n.d.
*m*-coumaric acid	0.36 ± 0.04 ^a^	n.d.	n.d.	10.48 ± 0.81 ^b^	0.25 ± 0.01 ^a^
*p*-coumaric acid	n.d.	1.69 ± 0.13 ^b^	0.040 ± 0.001 ^a^	3.69 ± 0.06 ^c^	1.00 ± 0.01 ^b^
Rosmarinic acid	226.98 ± 11.98 ^d^	n.d.	3.36 ± 0.20 ^a^	14.98 ± 1.03 ^c^	6.21 ± 0.36 ^b^
Chlorogenic acid	189.98 ± 12.27 ^c^	n.d.	1.59 ± 0.11 ^a^	25.12 ± 2.01 ^b^	1.02 ± 0.01 ^a^
Neochlorogenic acid	67.98 ± 3.14 ^d^	5.98 ± 0.04 ^b^	54.23 ± 2.23 ^c^	3.37 ± 0.24 ^a^	3.25 ± 0.02 ^a^
4-*O*-caffeoylquinic acid	12.69 ± 1.07 ^c^	n.d.	4.12 ± 0.22 ^b^	1.11 ± 0.01 ^a^	n.d.
Coumarin derivatives (μg/g of dry extract)					
Coumarin	79.87 ± 3.32 ^b^	n.d.	n.d.	5.31 ± 0.23 ^a^	n.d.
*m*-hydroxycoumarin	n.d.	2.64 ± 0.11 ^a^	19.95 ± 1.12 ^b^	n.d.	n.d.
*p*-hydroxycoumarin	2.21 ± 0.01 ^a^	n.d.	6.99 ± 0.21 ^b^	1.98 ± 0.13 ^a^	n.d.
7- hydroxycoumarin	5.69 ± 0.13 ^c^	n.d.	3.21 ± 0.13 ^b^	0.55 ± 0.07 ^a^	n.d.
Osthol	6.19 ± 0.12 ^b^	n.d.	n.d.	0.010 ± 0.00 ^a^	n.d.
Phenolic derivative (μg/g of dry extract)					
Eugenol	0.16 ± 0.00 ^a^	n.d.	0.69 ± 0.04 ^b^	n.d.	n.d.
Furanocoumarin derivatives (μg/g of dry extract)					
Isopimpinellin	n.d.	n.d.	n.d.	0.99 ± 0.06	n.d.
Xanthotoxin	n.d.	2.21 ± 0.13 ^a^	4.46 ± 0.21 ^b^	n.d.	n.d.
Xanthotoxol	n.d.	n.d.	5.69 ± 0.13	n.d.	n.d.
Flavanone derivatives (μg/g of dry extract)					
2′-hydroxyflavanone	1.36 ± 0.08	n.d.	n.d.	n.d.	n.d.
7-hydroxyflavanone	2.36 ± 0.10 ^c^	0.32 ± 0.01 ^a^	n.d.	0.99 ± 0.05 ^b^	3.32 ± 0.11 ^c^
4′-methoxyflavanone	2.21 ± 0.10 ^b^	n.d.	n.d.	0.18 ± 0.01 ^a^	n.d.
Naringin	2.17 ± 0.11	n.d.	n.d.	n.d.	n.d.
Flavone derivatives (μg/g of dry extract)					
Apigenin	150.98 ± 9.45 ^c^	0.21 ± 0.01 ^a^	n.d.	n.d.	6.77 ± 0.21 ^b^
Apigenin-7-*O*-glucoside	15.83 ± 0.26 ^b^	0.12 ± 0.01 ^a^	n.d.	n.d.	n.d.
Luteolin	203.36 ± 19.50 ^d^	0.36 ± 0.02 ^a^	n.d.	2.39 ± 0.10 ^b^	7.13 ± 0.41 ^c^
Luteolin-7-*O*-glucoside	150.98 ± 9.26 ^c^	0.21 ± 0.01 ^a^	n.d.	n.d.	6.77 ± 0.18 ^b^
Flavonol derivatives (μg/g of dry extract)					
Isorhamnetin	4.16 ± 0.23 ^b^	n.d.	n.d.	1.03 ± 0.01 ^a^	3.17 ± 0.18 ^b^
Quercetin	63.25 ± 2.49 ^d^	2.21 ± 0.14 ^a^	n.d.	40.21 ± 3.14 ^c^	12.11 ± 0.92 ^b^
Quercetin-3-*O*-rhamnoside	190.56 ± 12.34 ^e^	12.29 ± 1.09 ^c^	8.27 ± 0.41 ^d^	0.69 ± 0.05 ^b^	0.21 ± 0.01 ^a^
Quercetin-3-*O*-rutinoside	3.32 ± 0.11 ^b^	n.d.	n.d.	0.89 ± 0.05 ^a^	n.d.
Quercetin-3-*O*-galactoside	1.14 ± 0.01 ^b^	n.d.	n.d.	0.17 ± 0.01 ^a^	0.140 ± 0.001 ^a^
Myricetin-3-*O*-galactoside	30.21 ± 2.15 ^b^	n.d.	n.d.	0.14 ± 0.01 ^a^	n.d.
Myricetin-3-*O*-rhamnoside	36.15 ± 2.21 ^b^	n.d.	n.d.	n.d.	1.11 ± 0.01 ^a^
Kaempferol	n.d.	n.d.	n.d.	n.d.	1.06 ± 0.10
Kaempferol-3-*O*-rhamnoside	0.21 ± 0.01 ^a^	n.d.	n.d.	0.49 ± 0.03 ^b^	n.d.
Catechins and procyanidins (μg/g of dry extract)					
Procyanidin-B2	n.d.	0.21 ± 0.01 ^a^	n.d.	n.d.	1.42 ± 0.04 ^b^
(−)-Epicatechin	24.59 ± 1.01 ^c^	12.24 ± 0.63 ^b^	3.21 ± 0.14 ^a^	n.d.	n.d.
(Di)Terpenes (μg/g of dry extract)					
Carnosic acid	104.28 ± 9.81 ^c^	11.47 ± 0.69 ^b^	719.56 ± 53.27 ^d^	210.02 ± 99.80 ^c^	2.65 ± 0.13 ^a^
Carnosol	0.32 ± 0.02 ^a^	94.21 ± 6.21 ^b^	569.98 ± 42.14 ^d^	314.25 ± 2.21 ^c^	90.48 ± 3.28 ^b^

Data collections were obtained via UPLC-MS/MS and standardized at two decimal places. The data represent means ± standard deviation (SD) of six independent studies. Means and SDs preceded by distinct letters in a category significantly vary, as indicated by Tukey’s test (*p* < 0.05). The initials n.d. represent data for which the associated signals were not detectable.

**Table 3:** Antioxidant potential of the five *Salvia fruticosa* fractions, as calculated using the DPPH⋅ and the FRAP assays.

Extract		SFW1	SFDE	SFEA	SFB	SFW2
DPPH·	*EC*_50_ (mg dry extract/mg DPPH·)	0.34 ± 0.02	0.08 ± 0.01	0.28 ± 0.04	0.30 ± 0.01	0.47 ± 0.01
	AE	2.96	11.79	3.59	3.37	2.12
FRAP	μmol AAE/g	2432.30 ± 185.96	3034.28 ± 76.11	3880.82 ± 62.38	2499.06 ± 109.30	1165.42 ± 149.29
	μmol TEAC/g	2610.12 ± 96.27	3216.10 ± 56.97	4229.90 ± 213.91	2677.32 ± 215.68	1237.83 ± 107.97

Findings are displayed as average ± standard deviation.

## Data Availability

Data are contained within the article.

## Supplementary Materials

The following supporting information can be downloaded at: https://www.mdpi.com/article/10.3390/plants12183191/s1, Table S1: Dunnett’s multiple comparisons test conducted for the DCF-DA assay; Table S2: Dunnett’s multiple comparisons test conducted for the extracts’ cytotoxicity assay; Table S3: Dunnett’s multiple comparisons test conducted for the extracts’ anti-neurotoxic ability assay; Table S4: Multiple reaction monitoring conditions for polyphenolic acids and flavonoids used for content quantification in *S. fruticosa* via UPLC-MS/MS analysis; Table S5: The limits of detection (LOD) and quantification (LOQ), linearity, precision, and accuracy results for the screened polyphenolic compounds contained in *S. fruticosa*; Figure S1: Selected ion recording (SIR) (centroid acquisition) spectra of the fractions of methanolic extract; Figure S2: Effect of higher concentrations of the five SF methanolic partitions on ROS levels in the presence of H_2_O_2_ in SH-SY5Y cells; Figure S3: The chromatograms of the polyphenolic standards in the MRM mode, ionised with both positive and negative electron spray ionisation (±-ESI).
