# Supplementary material for: Chemical Profiling and Antioxidant and Anti-Amyloid Capacities of Salvia fruticosa Extracts from Greece
Source: Plants (Basel). 2023 Sep 6;12(18):3191. doi: 10.3390/plants12183191 (PMC10535607; doi:10.3390/plants12183191)
Supplement: Supplementary file 1 [file plants-12-03191-s001.zip › plants-2538509-supplementary.pdf]

Table S1: Dunnett's multiple comparisons test conducted for the DCF-DA assay. The presence of cellular ROS in control cells treated with 50 µM H_2O_2 only was compared with the presence of cellular ROS in cells treated with various concentrations of each extract and 50 µM H_2O_2 as well. *** refers to $p < 0.001$ statistical significance, compared with control cells treated with 50 µm H_2O_2 only.

Dunnett's multiple comparisons tests	Mean Difference	95.00% CI of difference	Below threshold?	Summary	Adjusted P Value
Control vs. Untreated	305.8	191.7 to 419.9	Yes	***	<.001
Control vs. 500 µM Trolox	325.4	211.3 to 439.4	Yes	***	<.001
Control vs. SFW1 200 µg/mL	313.4	199.3 to 427.5	Yes	***	<.001
Control vs. SFW1 100 µg/mL	332.9	218.8 to 447.0	Yes	***	<.001
Control vs. SFW1 50 µg/mL	332.4	218.3 to 446.5	Yes	***	<.001
Control vs. SFW1 2 µg/mL	266.5	152.5 to 380.6	Yes	***	<.001
Control vs. SFDE 200 µg/mL	218.2	104.2 to 332.3	Yes	***	<.001
Control vs. SFDE 100 µg/mL	291.6	177.5 to 405.7	Yes	***	<.001
Control vs. SFDE 50 µg/mL	328.5	214.5 to 442.6	Yes	***	<.001
Control vs. SFDE 2 µg/mL	331	216.9 to 445.0	Yes	***	<.001
Control vs. SFEA 200 µg/mL	289.2	175.1 to 403.3	Yes	***	<.001
Control vs. SFEA 100 µg/mL	322.6	208.5 to 436.7	Yes	***	<.001
Control vs. SFEA 50 µg/mL	336.3	222.2 to 450.4	Yes	***	<.001
Control vs. SFEA 2 µg/mL	321.7	207.6 to 435.8	Yes	***	<.001
Control vs. SFB 200 µg/mL	328.2	214.1 to 442.3	Yes	***	<.001
Control vs. SFB 100 µg/mL	341	226.9 to 455.1	Yes	***	<.001
Control vs. SFB 50 µg/mL	342.5	228.4 to 456.6	Yes	***	<.001
Control vs. SFB 20 µg/mL	269.2	155.1 to 383.2	Yes	***	<.001
Control vs. SFW2 200 µg/mL	307.6	193.6 to 421.7	Yes	***	<.001
Control vs. SFW2 100 µg/mL	335	220.9 to 449.1	Yes	***	<.001
Control vs. SFW2 50 µg/mL	335.3	221.2 to 449.4	Yes	***	<.001
Control vs. SFW2 20 µg/mL	219.6	105.5 to 333.7	Yes	***	<.001

Table S2: Dunnett's multiple comparisons test conducted for the extracts' cytotoxicity assay. The viability of untreated control cells was compared with the viability of cells treated with various concentrations of each extract. ** refers to $p < 0.01$, and *** to $p < 0.001$ statistical significance, compared with untreated cells (control).

Dunnett's multiple comparisons tests	Mean Difference	95.00% CI of difference	Below threshold?	Summary	Adjusted P Value
Control vs. SFW1 200 µg/mL	32.56	11.73 to 53.40	Yes	***	<.001
Control vs. SFW1 100 µg/mL	19.39	-1.444 to 40.23	No	ns	0.085
Control vs. SFW1 50 µg/mL	15.6	-5.234 to 36.44	No	ns	0.276
Control vs. SFW1 20 µg/mL	9.391	-11.44 to 30.23	No	ns	0.893
Control vs. SFDE 100 µg/mL	26.57	5.731 to 47.40	Yes	**	0.004
Control vs. SFDE 50 µg/mL	17.9	-2.939 to 38.73	No	ns	0.14
Control vs. SFDE 20 µg/mL	12.67	-8.169 to 33.50	No	ns	0.554
Control vs. SFEA 400 µg/mL	96.24	75.40 to 117.1	Yes	***	<.001
Control vs. SFEA 200 µg/mL	3.108	-17.73 to 23.94	No	ns	>.999
Control vs. SFEA 100 µg/mL	2.183	-18.65 to 23.02	No	ns	>.999
Control vs. SFEA 50 µg/mL	-5.154	-25.99 to 15.68	No	ns	0.999
Control vs. SFEA 20 µg/mL	-0.6316	-21.47 to 20.20	No	ns	>.999
Control vs. SFB 800 µg/mL	28.28	7.446 to 49.12	Yes	**	0.002
Control vs. SFB 400 µg/mL	-15.24	-36.08 to 5.593	No	ns	0.304
Control vs. SFB 200 µg/mL	-0.6466	-21.48 to 20.19	No	ns	>.999
Control vs. SFB 100 µg/mL	-6.944	-27.78 to 13.89	No	ns	0.988
Control vs. SFB 50 µg/mL	-8.877	-29.71 to 11.96	No	ns	0.929
Control vs. SFB 20 µg/mL	-7.002	-27.84 to 13.83	No	ns	0.988
Control vs. SFW2 800 µg/mL	43.99	23.16 to 64.83	Yes	***	<.001
Control vs. SFW2 400 µg/mL	20.82	-0.01603 to 41.65	No	ns	0.05
Control vs. SFW2 200 µg/mL	-1.742	-22.58 to 19.09	No	ns	>.999
Control vs. SFW2 100 µg/mL	-0.1266	-20.96 to 20.71	No	ns	>.999
Control vs. SFW2 50 µg/mL	9.363	-11.47 to 30.20	No	ns	0.896
Control vs. SFW2 20 µg/mL	15.37	-5.469 to 36.20	No	ns	0.294

Table S3: Dunnett's multiple comparisons test conducted for the extracts' anti-neurotoxic ability assay. The viability of cells treated with 25 µM A$\beta_{25\text{-}35}$ only was compared with the viability of cells treated with various concentrations of each extract and 25 µM A$\beta_{25\text{-}35}$ as well. * refers to $p < 0.05$ statistical significance, compared with cells treated only with 25 µM A$\beta_{25\text{-}35}$.

Dunnett's multiple comparisons tests	Mean Difference	95.00% CI of difference	Below threshold?	Summary	Adjusted P Value
A$\beta_{25\text{-}35}$ only vs. SFW1 100 µg/mL	-2.66	-16.10 to 10.78	No	ns	>.999
A$\beta_{25\text{-}35}$ only vs. SFW1 50 µg/mL	-5.118	-18.56 to 8.322	No	ns	0.975
A$\beta_{25\text{-}35}$ only vs. SFW1 20 µg/mL	-14.01	-27.45 to -0.5722	Yes	*	0.035
A$\beta_{25\text{-}35}$ only vs. SFW1 2 µg/mL	-2.002	-15.44 to 11.44	No	ns	>.999
A$\beta_{25\text{-}35}$ only vs. SFDE 50 µg/mL	13.76	0.3182 to 27.20	Yes	*	0.041
A$\beta_{25\text{-}35}$ only vs. SFDE 20 µg/mL	11.1	-2.336 to 24.54	No	ns	0.175
A$\beta_{25\text{-}35}$ only vs. SFDE 2 µg/mL	-14.14	-27.58 to -0.7022	Yes	*	0.033
A$\beta_{25\text{-}35}$ only vs. SFEA 200 µg/mL	5.492	-7.948 to 18.93	No	ns	0.952
A$\beta_{25\text{-}35}$ only vs. SFEA 100 µg/mL	4.472	-8.968 to 17.91	No	ns	0.988
A$\beta_{25\text{-}35}$ only vs. SFEA 50 µg/mL	-4.822	-18.26 to 8.618	No	ns	0.985
A$\beta_{25\text{-}35}$ only vs. SFEA 20 µg/mL	-3.346	-16.79 to 10.09	No	ns	0.999
A$\beta_{25\text{-}35}$ only vs. SFEA 2 µg/mL	-13.73	-27.17 to -0.2902	Yes	*	0.042
A$\beta_{25\text{-}35}$ only vs. SFB 400 µg/mL	-9.196	-22.64 to 4.244	No	ns	0.396
A$\beta_{25\text{-}35}$ only vs. SFB 200 µg/mL	-14.46	-27.90 to -1.016	Yes	*	0.027
A$\beta_{25\text{-}35}$ only vs. SFB 100 µg/mL	-14.04	-27.48 to -0.6042	Yes	*	0.035
A$\beta_{25\text{-}35}$ only vs. SFB 50 µg/mL	-7.914	-21.35 to 5.526	No	ns	0.607
A$\beta_{25\text{-}35}$ only vs. SFB 20 µg/mL	-3.158	-16.60 to 10.28	No	ns	0.999
A$\beta_{25\text{-}35}$ only vs. SFB 2 µg/mL	-3.05	-16.49 to 10.39	No	ns	>.999
A$\beta_{25\text{-}35}$ only vs. SFW2 400 µg/mL	2.982	-10.46 to 16.42	No	ns	>.999
A$\beta_{25\text{-}35}$ only vs. SFW2 200 µg/mL	2.09	-11.35 to 15.53	No	ns	>.999
A$\beta_{25\text{-}35}$ only vs. SFW2 100 µg/mL	-4.562	-18.00 to 8.878	No	ns	0.988
A$\beta_{25\text{-}35}$ only vs. SFW2 50 µg/mL	-2.906	-16.35 to 10.53	No	ns	>.999
A$\beta_{25\text{-}35}$ only vs. SFW2 20 µg/mL	-3.242	-16.68 to 10.20	No	ns	0.999
A$\beta_{25\text{-}35}$ only vs. SFW2 2 µg/mL	5.276	-8.164 to 18.72	No	ns	0.967

Table S4: Multiple Reaction Monitoring conditions for polyphenolic acids and flavonoids used for content quantification in *S. fruticosa* via UPLC-MS/MS analysis.

Polyphenolic compound	Chemical formula	Molecular weight	[M-H]± (m/z)	MS^2 fragments (m/z)	Cone voltage (V)	Collision energy (eV)	Retention time (R_t)
**Benzoic acid derivatives**							
m-hydroxy benzoic acid	$C_7H_6O_3$	138.13	137.05	92.90	22	10	2.34
p-hydroxy benzoic acid	$C_7H_6O_3$	138.12	136.95	65.00<br>93.00	23	25<br>13	1.88
Protocatechuic acid	$C_7H_6O_4$	154.12	152.95	108.95	25	13	1.64
Vanillin	$C_8H_8O_3$	152.15	151.00	92.20<br>136.0	22	20<br>15	2.23
p-hydroxy benzaldehyde	$C_7H_6O_2$	122.13	120.95	91.85	12	20	4.69
Gentisic acid	$C_7H_6O_4$	154.13	153.05	108.20	13	25	4.28
**Gallic acid derivatives**							
Gallic acid	$C_7H_6O_5$	170.12	168.95	78.98<br>124.95	23	22<br>15	1.37
Ethyl gallate	$C_9H_{10}O_5$	198.18	197.05	124.00	15	25	4.66
Syringic acid	$C_9H_{10}O_5$	198.17	197.00	122.95<br>182.00	27	23<br>13	1.93
Ellagic acid	$C_{14}H_6O_8$	302.19	301.00	145.00<br>173.00	35	34<br>36	2.00
**Cinnamic acid derivatives**							
Ferulic acid	$C_{10}H_{10}O_4$	194.18	192.95	134.00<br>178.00	26	25<br>12	2.20
Ferulic acid ethyl ester	$C_{12}H_{14}O_4$	222.24	223.20	177.15	15	13	5.84
Ferulic acid methyl ester	$C_{11}H_{12}O_4$	208.22	209.05	177.15	16	5	5.48
Caffeic acid	$C_9H_8O_4$	180.16	178.95	134.95	25	13	1.89
Dihydro caffeic acid	$C_9H_{10}O_4$	182.17	181.05	137.05	22	12	4.39
trans-cinnamaldehyde	C_9H_8O	132.16	133.15	55.00	20	11	5.93
trans-cinnamyl alcohol	$C_9H_{10}O$	134.18	117.25	115.00	18	17	5.45
m-coumaric acid	$C_9H_8O_3$	164.16	163.10	118.90	20	12	4.81
p-coumaric acid	$C_9H_8O_3$	164.16	163.00	119.00	15	13	2.13
Rosmarinic acid	$C_{18}H_{16}O$	360.32	359.20	161.00<br>197.00	10	15<br>15	2.26
Chlorogenic acid	$C_{16}H_{18}O_9$	354.31	353.10	84.00<br>191.02	22	44<br>14	1.70
Neochlorogenic acid	$C_{16}H_{18}O_9$	354.31	353.15	135.18	19	36	2.50

4-*O*-caffeoylquinic acid	$C_{16}H_{18}O_9$	354.31	353.35	173.20	19	18	4.28
**Coumarin derivatives**							
Coumarin	$C_9H_6O_2$	146.15	147.01	91.00<br>102.90	17	12<br>17	5.44
m-hydroxycoumarin	$C_9H_6O_3$	162.15	161.15	133.00	17	13	5.26
p-hydroxycoumarin	$C_9H_6O_3$	162.15	163.00	121.00	39	13	5.04
7-hydroxycoumarin	$C_9H_6O_3$	162.15	163.15	107.20	20	21	4.97
Osthol	$C_{15}H_{16}O_3$	244.29	245.18	131.10<br>189.00	14<br>14	21<br>13	0.28
**Phenolic derivative**							
Eugenol	$C_{10}H_{12}O_2$	164.21	165.25	123.95	14	15	6.08
**Furanocoumarin derivatives**							
Isopimpinellin	$C_{13}H_{10}O_5$	246.22	247.15	217.10	27	24	5.93
Xanthotoxin	$C_{12}H_8O_4$	216.19	217.15	161.20<br>202.10	15	20<br>25	5.75
Xanthotoxol	$C_{11}H_6O_4$	202.17	203.15	131.15<br>147.05	22	18<br>21	5.09
**Flavanone derivatives**							
2'-hydroxyflavanone	$C_{15}H_{12}O_3$	240.27	239.00	119.30<br>93.10	40	25<br>16	3.18
7-hydroxyflavanone	$C_{15}H_{12}O_3$	240.27	239.05	135.20<br>91.15	41	25<br>23	3.42
4'-methoxyflavanone	$C_{16}H_{14}O_3$	254.29	255.15	240.00<br>161.30	31	17<br>22	3.78
Naringin	$C_{22}H_{32}O_{14}$	580.54	579.15	271.10<br>151.50	45	33<br>40	2.12
**Flavone derivatives**							
Apigenin	$C_{15}H_{10}O_5$	270.05	269.15	117.00	31	29	5.24
Apigenin-7-*O*-glucoside	$C_{21}H_{20}O_{10}$	432.38	431.15	268.35	35	22	2.15
Luteolin	$C_{15}H_{10}O_6$	286.25	285.13	133.25	24	37	4.99
Luteolin-7-*O*-glucoside	$C_{21}H_{20}O_{11}$	448.38	449.15	287.10	34	31	2.01
**Flavonol derivatives**							
Isorhamnetin	$C_{16}H_{12}O_7$	316.28	315.00	151.00<br>300.20	43	30<br>20	2.86
Quercetin	$C_{15}H_{10}O_7$	302.23	301.35	151.00<br>179.10	13	18<br>14	5.09
Quercetin-3-*O*-rhamnoside	$C_{21}H_{20}O_{11}$	448.38	447.01	271.00<br>300.00	43	47<br>28	2.14

Quercetin-3-*O*-rutinoside	$C_{27}H_{30}O_{16}$	610.53	609.10	300.0<br>271.00	47	39<br>65	1.92
Quercetin-3-*O*-galactoside	$C_{21}H_{20}O_{12}$	464.38	463.30	300.00<br>271.15	47	24<br>44	1.99
Myricetin-3-*O*-galactoside	$C_{21}H_{20}O_{13}$	480.38	479.05	271.10<br>287.10	48	39<br>44	1.87
Myricetin-3-*O*-rhamnoside	$C_{21}H_{20}O_{12}$	464.38	463.15	316.30<br>271.25	31	27<br>42	4.38
Kaempferol	$C_{15}H_{10}O_6$	286.25	285.25	151.00	30	14	5.32
Kaempferol-3-*O*-rutinoside	$C_{21}H_{20}O_{10}$	432.39	431.05	255.30<br>284.20	45	42<br>28	2.27
**Catechins and Procyanidins**							
Procyanidin-B2	$C_{30}H_{26}O_{12}$	578.53	577.00	125.05<br>289.30<br>407.35	29	32<br>23<br>24	2.30
(-)-Epicatechin	$C_{15}H_{14}O_6$	290.28	289.05	108.90<br>123.05	30	29<br>29	4.29
**(Di)terpenes**							
Carnosol	$C_{20}H_{26}O_4$	330.42	329.53	286.12	20	23	7.01
Carnosic acid	$C_{20}H_{28}O_4$	332.42	331.50	287.98	20	23	7.94

Table S5: The limit of detection (LOD), quantification (LOQ), linearity, precision, and accuracy results for the screened polyphenolic compounds contained in *S. fruticosa*. The calibration equations represent the peak area as a function of concentration in ppb. The intra- and inter-day experimental data concern data that have been collected from a six-day experiment, whereas the %recovery data are the means of three independent experiments.

Compound	Linear range (ppb)	LOD (ppb)	LOQ (ppb)	Calibration equation[a]	Correlation coefficient (r^2)	%RSD		%REC[d]
						(intra-day)[b]	(inter-day)[c]	
**Benzoic acid derivatives**								
m-hydroxybenzoic acid	13.39-499.21	13.39	40.58	y=42.16x+277.25	0.9997	1.00	2.06	99.9
p-hydroxybenzoic acid	3.01-499.50	3.01	14.20	y=36.87x-62.07	0.9991	1.15	2.21	98.8
Protocatechuic acid	0.66-504.50	0.66	14.70	y=34.24x-69.4	0.9995	1.25	2.65	86.3
Vanillin	2.87-335.00	2.87	5.62	y=0.67x-0.1	0.9999	0.98	0.95	100.4
p-hydroxy benzaldehyde	0.57-99.46	0.57	1.72	y=7.43x+36.81	0.9991	3.21	3.25.	95.6
Gentisic acid	7.06	7.06	21.40	y=3.40x+36.81	0.9994	2.47	2.6	100.3
**Gallic acid derivatives**								
Gallic acid	53.20-513.20	53.20	105.20	y=0.67x-1.5	0.9996	0.46	0.21	99.9
Ethyl gallate	1.21-108.36	1.21	3.69	y=21.07x+123.35	0.9999	1.06	1.33	97.5
Syringic acid	2.01-501.60	2.01	2.86	y=7.28x-2.7	0.9996	1.36	1.01	96.6
Ellagic acid	5.53-499.10	5.53	75.60	y=2.18x+7.4	0.9995	1.32	3.05	89.9
**Cinnamic acid derivatives**								
Ferulic acid	2.10-505.60	2.10	12. 17	y=19.02x-68.4	0.9992	0.7	2.45	102.6
Ferulic acid methyl ester	0.45-499.76	0.45	1.37	y=793.13x+4764.76	0.9999	2.21	2.98	83.4
Ferulic acid ethyl ester	0.11-498.50	0.11	0.32	y=2013.99x+16320.9	0.9994	3.21	3.69	93.6
Caffeic acid	1.21-500	1.21	1.25	y=92.95x+344.4	0.9995	1.01	2.21	100.1
Dihydrocaffeic acid	97.28-499.66	97.28	294.81	y=11.92+64.06	0.9998	0.97	1.14	94.4
trans-cinnamaldehyde	0.76-500.6	0.76	2.30	y=270.02x+832.24	0.9997	1.59	2.63	93.2
trans-cinnamyl alcohol	1.37-498.14	1.37	4.16	y=82.34x+552.83	0.9993	2.24	2.59	99.9

p-coumaric acid	0.65-497.30	0.65	1.55	y=52.84x+36.9	0.9997	1.7	1.94	93.2
m-coumaric acid	1.21-497.70	1.21	2.87	Y=70.92x+473.76	0.9996	1.64	2.24	97.8
Rosmarinic acid	2.32-499.50	2.32	2.56	y=7.03x+12.34	0.9996	1.3	3.02	86.9
Chlorogenic acid	3.48-495.60	3.48	4.76	y=25.02x+60.3	0.9991	1.35	1.98	87.4
Neochlorogenic acid	3.21-499.85	3.21	9.72	y=85.69x+39.87	0.9996	1.11	2.48	99.9
4-O-caffeoylquinic acid	2.29-496.34	2.29	6.93	y=15.9x+12.25	0.9963	1.21	2.19	98.7
**Coumarin derivatives**								
Coumarin	0.91-497.55	0.91	2.76	y=2370.35x+15986.5	0.9997	2.36	3.62	92.1
m-hydroxycoumarin	11.84-503.71	11.84	35.90	y=2.59x+22.96	0.9970	1.34	3.01	97.8
p-hydroxycoumarin	1.06-498.01	1.06	3.21	y=1104.02x+9957.32	0.9992	2.21	2.59	96.1
7- hydroxycoumarin	1.98-498.10	1.98	6.02	Y=62.10x+907.80	0.9990	1.94	2.46	99.24
Osthol	0.11-499.80	0.11	0.3	y=1772.94x+30399.4	0.9999	1.36	2.15	95.7
**Phenolic derivative**								
Eugenol	3.63-497.40	3.63	11.01	y=89.12x+643	0.9987	2.25	4.68	101.2
**Furanocoumarin derivatives**								
Psoralen	1.21-499.30	1.21	3.66	y=3573.86x+22526.4	0.9994	2.18	2.69	96.4
Isopimpinellin	0.45-96.16	0.45	14.85	y=2987.59x+3078.12	0.9975	3.21	4.12	98.4
Xanthotoxin	0.30-496.65	0.3	0.9	y=983.972x+13022.7	0.9996	2.69	3.62	101.2
Xanthotoxol	0.27-498.16	0.27	0.81	y=1276.71x+9756.89	0.9992	1.58	2.21	95.5
**Flavanone derivatives**								
2’-hydroxyflavanone	19.50-250.00	19.50	20.12	y=38.69x+22.5	0.9998	2.7	4.32	99.5
7-hydroxyflavanone	1.97-249.90	1.97	2.21	y=51.17x-73.6	1	2.63	1.42	98.9
4’-methoxyflavanone	2.21-250.00	2.21	3.89	y=83.54x+60.3	0.9999	2.89	1.87	93.6
Naringin	3.01-250.60	3.01	1.21	y=22.88x-43.3	0.9997	2.22	4.02	95.4

**Flavone derivatives**								
Apigenin	1.53-499.98	1.53	4.66	y=64.17x+735.75	0.9998	1.36	2.47	97.7
Apigenin-7-*O*-glucoside	1.87-125.30	1.87	4.42	y=6.17x+3.8	0.9998	3.48	2.54	95.8
Luteolin	5.85-499.37	5.85	17.73	y=10.02x+123.29	0.9997	1.11	2.21	99.1
Luteolin-7-*O*-glucoside	2.21-250.10	2.21	2.22	y=51.52x-89.9	0.9998	3.64	3.22	89.2
**Flavonol derivatives**								
Isorhamnetin	14.01-251.1	14.01	2.31	y=6.08x-15.4	0.9992	2.48	1.18	100.1
Quercetin	1.98-250.06	1.98	6.00	y=32.48x-11.28	1	3.21	4.68	98.7
Quercetin-3-*O*-rhamnoside	1.02-250.60	1.02	4.21	y=60.83x-38.6	0.9999	2.21	3.01	99.8
Quercetin-3-*O*-rutinoside	1.40-251.30	1.40	4.32	y=97.74x+109.7	0.9999	1.35	1.89	87.4
Quercetin-3-*O*-galactoside	6.32-249.90	6.32	3.21	y=3.97x+0.5	0.9998	2.14	1.37	96.3
Myricetin-3-*O*-rhamnoside	3.12-500.00	3.12	9.47	y=17.99x+80.65	0.9997	1.10	2.01	100.6
Myricetin-3-*O*-galactoside	0.85-251.20	0.85	2.12	y=26.38x-31.8	0.9997	1.78	1.65	100.2
Kaempferol	10.61-499.28	10.61	32.16	y=2.59543x+14.47	0.9995	2.32	2.69	89.9
Kaempferol-3-*O*-rutinoside	0.76-250.00	0.76	1.21	y=25.73x+73.7	0.9997	1.36	2.21	91.2
**Catechins and Procyanidins**								
Procyanidin-B2	2.04-498.94	2.04	6.18	y=10.76x+42.68	0.9998	2.36	3.12	96.3
(-)-Epicatechin	7.90-498.50	7.90	29.94	y=7.50x+84.57	0.9918	1.26	2.36	97.8
**(Di)terpenes**								
Carnosic acid	2.35-499.99	2.35	7.12	y=45x+69.87	0.9999	1.96	2.68	89.8
Carnosol	1.12-489-69	1.12	3.39	y.36.14x-23.64	1	2.21	2.98	93.6

Figure S1: Selected ion recording (SIR) (centroid acquisition) spectra of the fractions of methanolic extract: (A) the aqueous (SFW1), (B) diethyl ether (SFDE), (C) ethyl acetate (SFEA), (D) butanolic (organic phase) (SFB) and (E) aqueous-phase of butanol (SFW2) of *S. fruticosa*. Scanning (m/z; 120–650) was carried out utilizing collisions energies and m/z in the negative and positive electrospray ionization (ESI±) mode in accordance with the collision energy as presented in Table S4: 1) *trans*-cinnamyl alcohol, 2) *p*-hydroxy benzaldehyde, 3) *m*-hydroxybenzoic acid, 4) coumarin, 5) vanillin, 6) protocatechuic acid, 7) gentisic acid, 8) *p*-hydroxy coumarin, 9) *m*-coumaric acid, 10) 7-hydroxy coumarin, 11) eugenol, 12) gallic acid, 13) caffeic acid, 14) dihydro caffeic acid, 15) ferulic acid, 16) syringic acid, 17) ethyl gallate, 18) ferulic acid ethyl ester, 19) 2'-hydroxyflavanone, 20) 7-hydroxyflavanone, 21) osthol, 22) 4'-methoxyflavanone, 23) apigenin, 24) luteolin, 25) (-)-epicatechin, 26) ellagic acid, 27) quercetin, 28) isorhamnetin, 29) carnosol, 30) carnosic acid, 31) chlorogenic acid, 32) neochlorogenic acid, 33) 4-*O*-caffeoylquinic acid, 34) rosmarinic acid, 35) kaempferol-3-*O*-rutinoside, 36) apigenin-7-*O*-glucoside, 37) quercetin-3-*O*-rhamnoside, 38) luteolin-7-*O*-glucoside, 39) myricetin-3-*O*-rhamnoside, 40) quercetin-3-*O*-galactoside, 41) myricetin-3-*O*-galactoside, 42) naringin, 43) quercetin-3-*O*-rutinoside, 44) *trans*-cinnamaldehyde, 45) *p*-hydroxy benzoic acid, 46) *m*-hydroxy coumarin, 47) *p*-coumaric acid, 48) ferulic acid methyl ester, 49) xanthotoxin, 50) procyanidin-B2, 51) xanthotoxol, 52) isopimpinellin, 53) kaempferol.

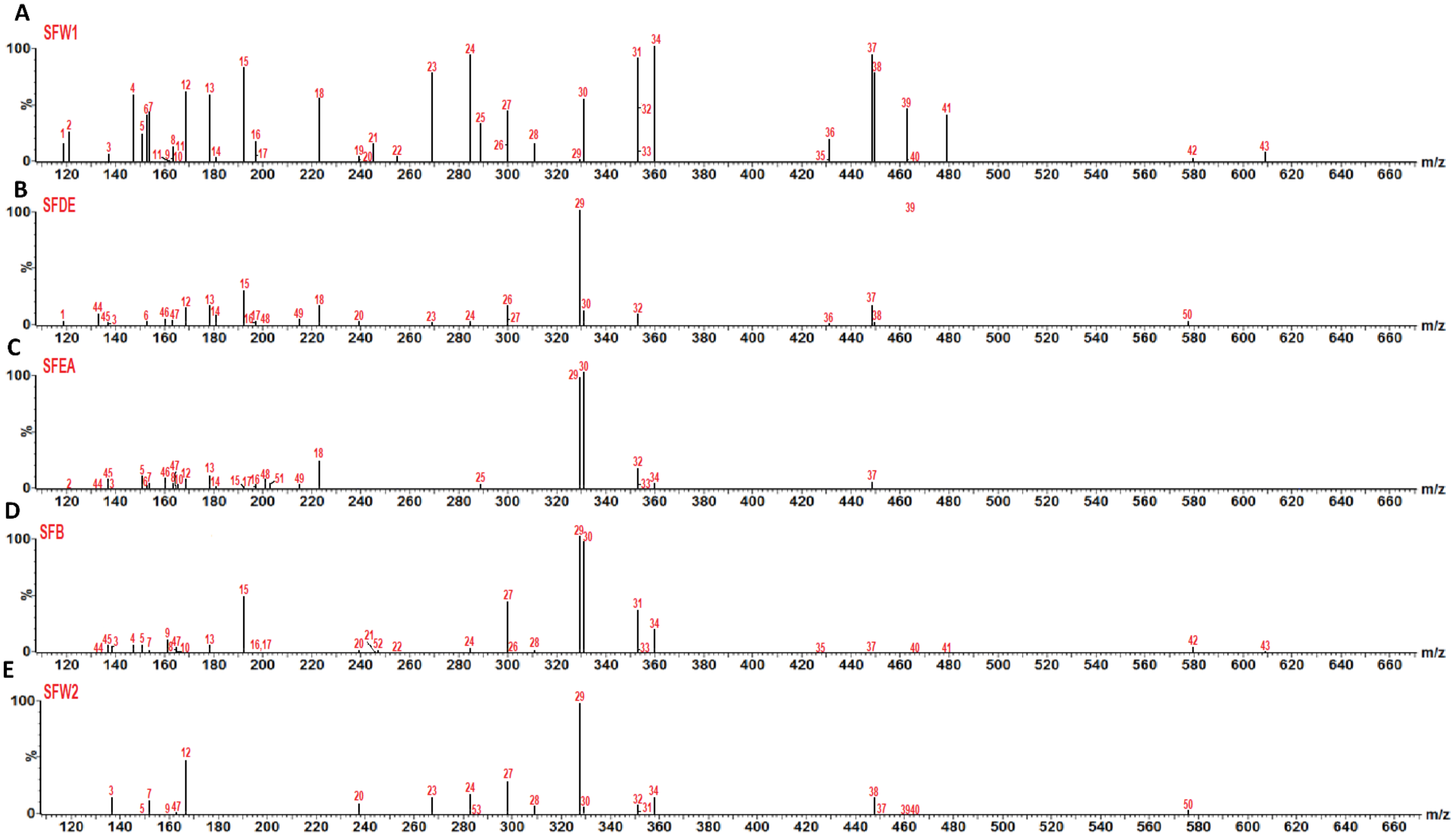

A
SFW1
B
SFDE
C
SFEA
D
SFB
E
SFW2
100
%
0
m/z
120 140 160 180 200 220 240 260 280 300 320 340 360 380 400 420 440 460 480 500 520 540 560 580 600 620 640 660

Figure S2: Effect of higher concentrations of the five SF methanolic partitions on ROS levels in the presence of H_2O_2 in SH-SY5Y cells. Error bars depict the standard error of the mean of four independent experiments. Statistically significant differences between group means were determined by one-way analysis of variance (F (12, 39) = 13.09, p<0.001). Post hoc Dunnett's multiple comparison tests followed, to compare the control with each different extract's treatment. * refers to p < 0.05, ** to p < 0.01, and *** to p < 0.001 statistical significance, compared with control cells that were treated with 50 μm H_2O_2 only.

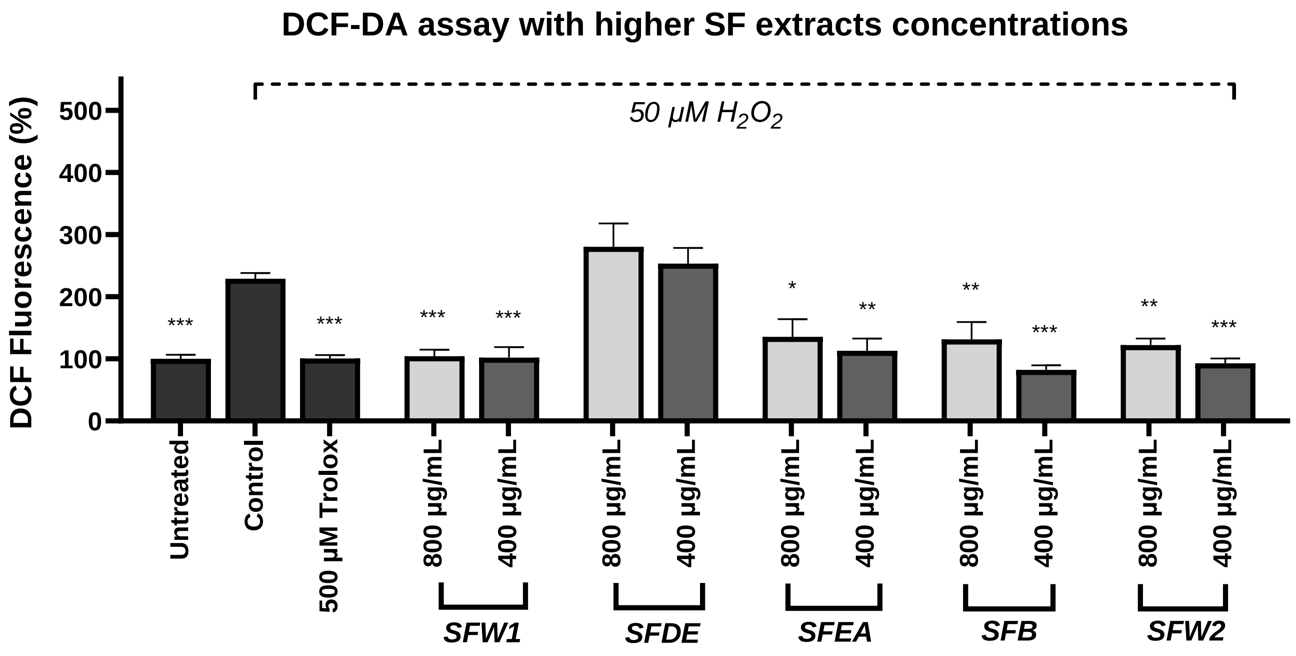

Figure S3: The chromatograms of the polyphenolic standards in the MRM mode, ionised with both positive and negative electron spray ionisation (±-ESI). The quantification signal is denoted into the spectra.

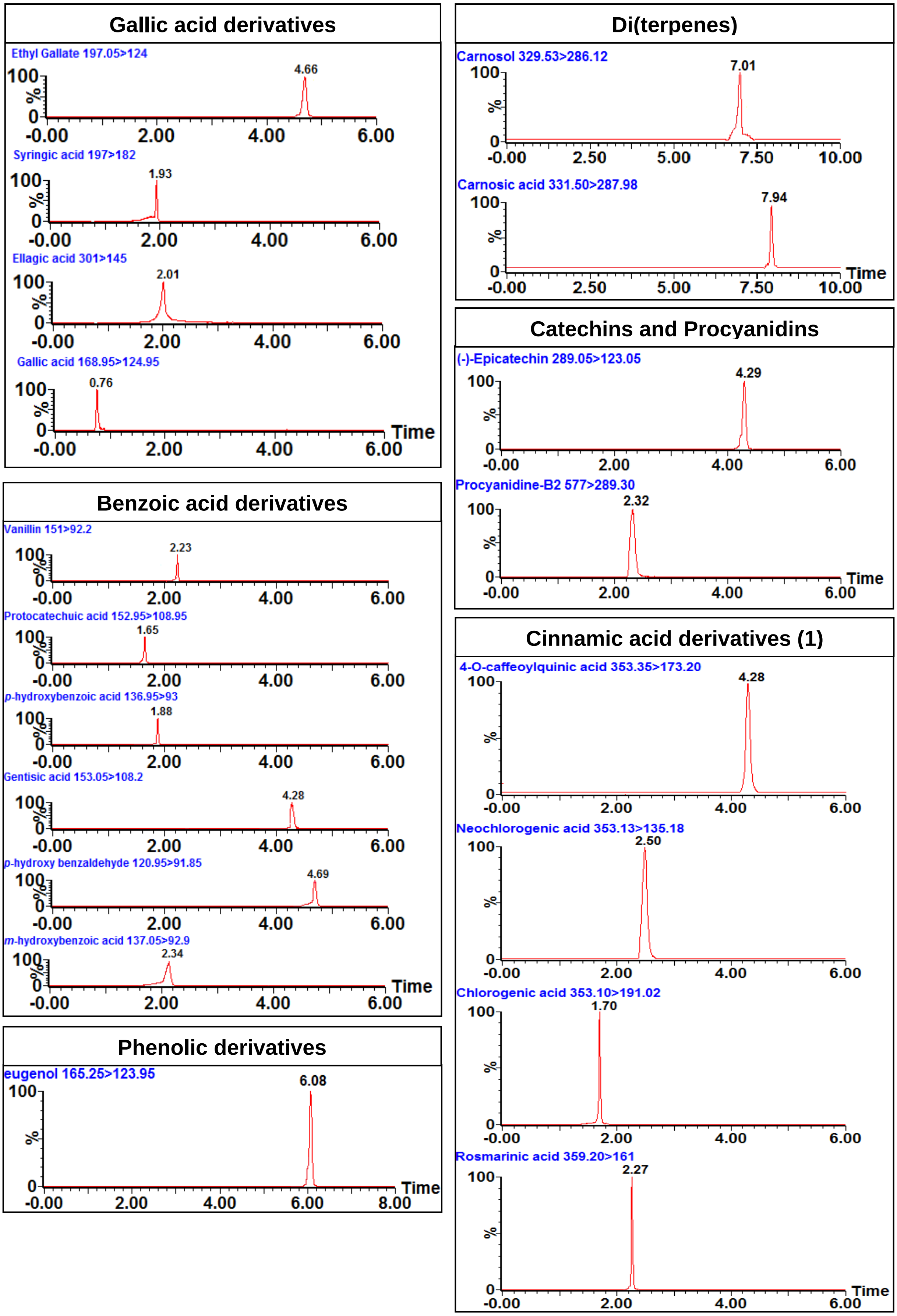

Gallic acid derivatives
Ethyl Gallate 197.05>124
4.66
Syringic acid 197>182
1.93
Ellagic acid 301>145
2.01
Gallic acid 168.95>124.95
0.76
Time
Benzoic acid derivatives
Vanillin 151>92.2
2.23
Protocatechuic acid 152.95>108.95
1.65
p-hydroxybenzoic acid 136.95>93
1.88
Gentisic acid 153.05>108.2
4.28
p-hydroxy benzaldehyde 120.95>91.85
4.69
m-hydroxybenzoic acid 137.05>92.9
2.34
Time
Phenolic derivatives
eugenol 165.25>123.95
6.08
Time
Di(terpenes)
Carnosol 329.53>286.12
7.01
Carnosic acid 331.50>287.98
7.94
Time
Catechins and Procyanidins
(-)-Epicatechin 289.05>123.05
4.29
Procyanidine-B2 577>289.30
2.32
Time
Cinnamic acid derivatives (1)
4-O-caffeoylquinic acid 353.35>173.20
4.28
Neochlorogenic acid 353.13>135.18
2.50
Chlorogenic acid 353.10>191.02
1.70
Rosmarinic acid 359.20>161
2.27
Time

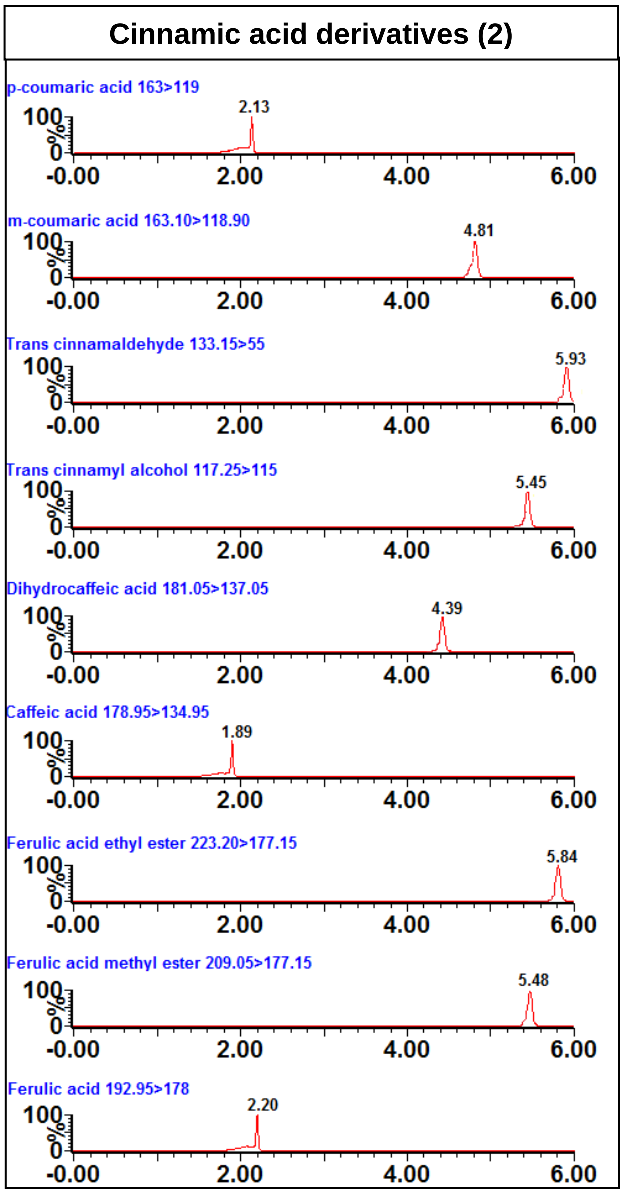
Cinnamic acid derivatives (2)
p-coumaric acid 163>119
2.13
m-coumaric acid 163.10>118.90
4.81
Trans cinnamaldehyde 133.15>55
5.93
Trans cinnamyl alcohol 117.25>115
5.45
Dihydrocaffeic acid 181.05>137.05
4.39
Caffeic acid 178.95>134.95
1.89
Ferulic acid ethyl ester 223.20>177.15
5.84
Ferulic acid methyl ester 209.05>177.15
5.48
Ferulic acid 192.95>178
2.20
100
%
0
-0.00
2.00
4.00
6.00

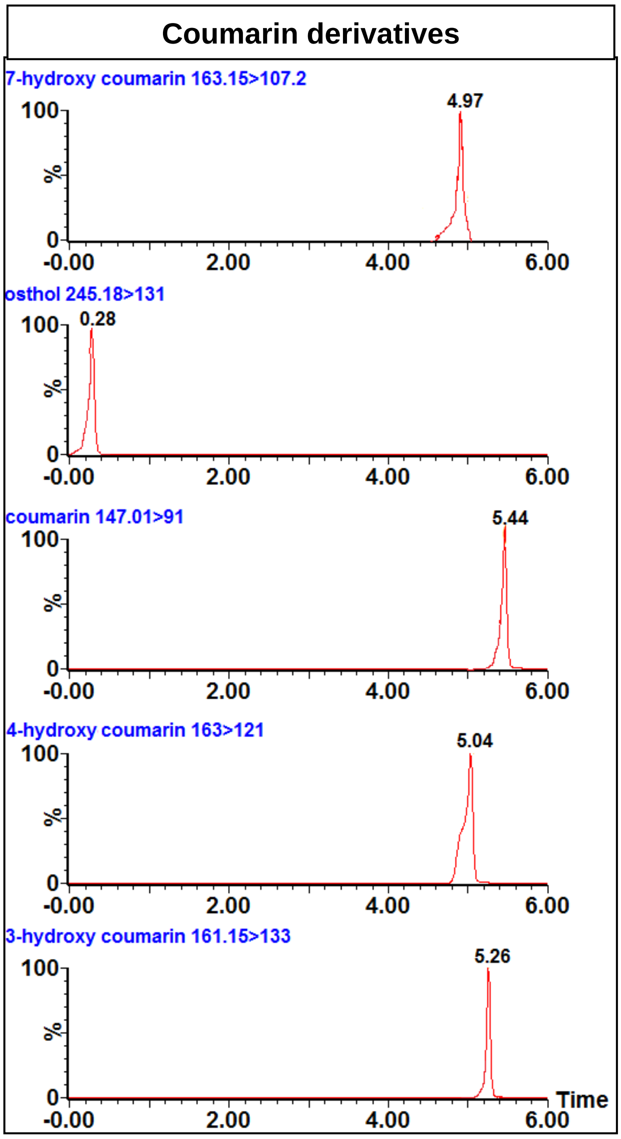
Coumarin derivatives
7-hydroxy coumarin 163.15>107.2
4.97
osthol 245.18>131
0.28
coumarin 147.01>91
5.44
4-hydroxy coumarin 163>121
5.04
3-hydroxy coumarin 161.15>133
5.26
100
%
0
-0.00
2.00
4.00
6.00
Time

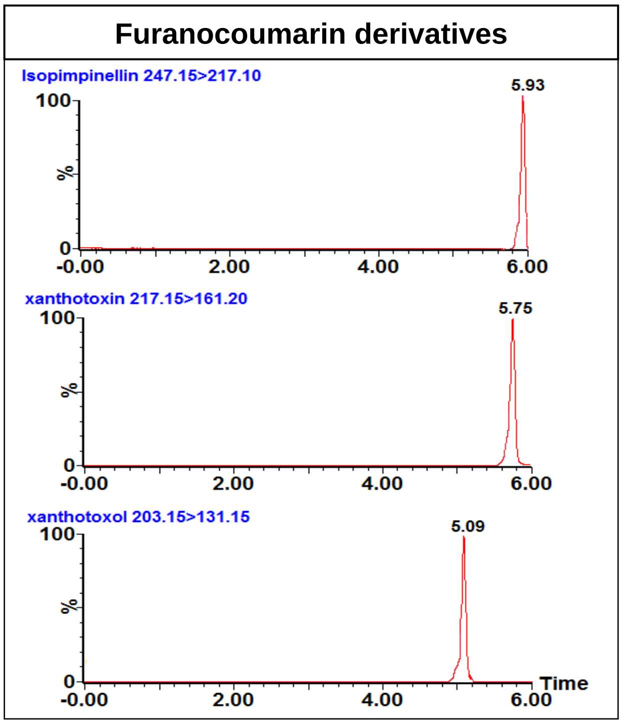
Furanocoumarin derivatives
Isopimpinellin 247.15>217.10
5.93
xanthotoxin 217.15>161.20
5.75
xanthotoxol 203.15>131.15
5.09
100
%
0
-0.00
2.00
4.00
6.00
Time

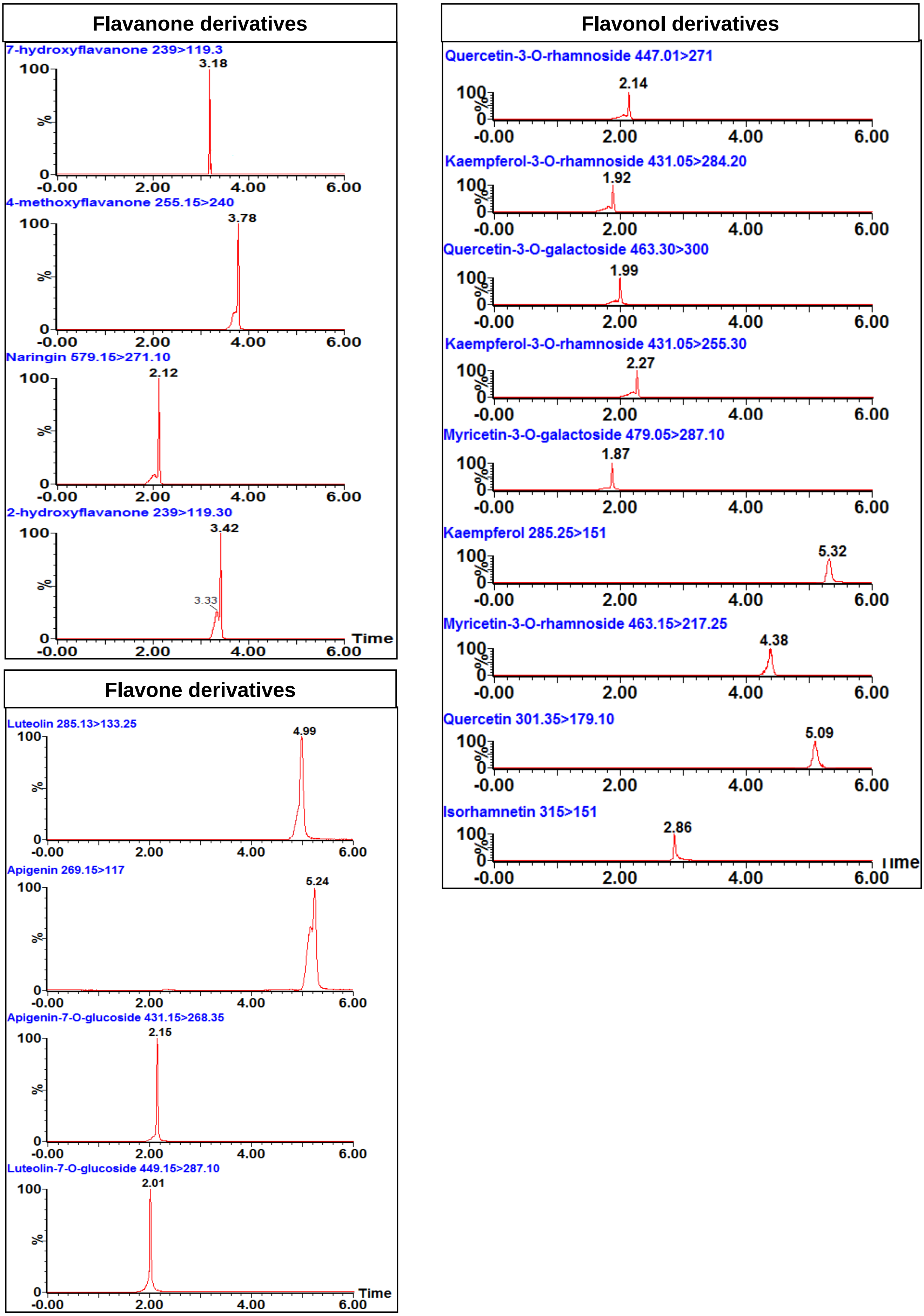
Flavanone derivatives
7-hydroxyflavanone 239>119.3
3.18
4-methoxyflavanone 255.15>240
3.78
Naringin 579.15>271.10
2.12
2-hydroxyflavanone 239>119.30
3.42
3.33
Time
Flavone derivatives
Luteolin 285.13>133.25
4.99
Apigenin 269.15>117
5.24
Apigenin-7-O-glucoside 431.15>268.35
2.15
Luteolin-7-O-glucoside 449.15>287.10
2.01
Time
Flavonol derivatives
Quercetin-3-O-rhamnoside 447.01>271
2.14
Kaempferol-3-O-rhamnoside 431.05>284.20
1.92
Quercetin-3-O-galactoside 463.30>300
1.99
Kaempferol-3-O-rhamnoside 431.05>255.30
2.27
Myricetin-3-O-galactoside 479.05>287.10
1.87
Kaempferol 285.25>151
5.32
Myricetin-3-O-rhamnoside 463.15>217.25
4.38
Quercetin 301.35>179.10
5.09
Isorhamnetin 315>151
2.86
100
%
0
-0.00
2.00
4.00
6.00
